# FMNL1 and mDia1 promote efficient T cell migration through complex environments via distinct mechanisms

**DOI:** 10.3389/fimmu.2024.1467415

**Published:** 2024-10-04

**Authors:** Ashton L. Sigler, Scott B. Thompson, Logan Ellwood-Digel, Adithan Kandasamy, Mary J. Michaels, Dean Thumkeo, Shuh Narumiya, Juan C. Del Alamo, Jordan Jacobelli

**Affiliations:** ^1^ Department of Immunology & Microbiology and Barbara Davis Research Center, University of Colorado School of Medicine, Aurora, CO, United States; ^2^ Department of Mechanical Engineering, University of Washington, Seattle, WA, United States; ^3^ Department of Drug Discovery Medicine, Kyoto University Graduate School of Medicine, Kyoto, Japan; ^4^ Division of Cardiology, University of Washington, Seattle, WA, United States

**Keywords:** T cell, formins, cell migration, motility, FMNL1, mDia1, DIAPH1, FRL1

## Abstract

Lymphocyte trafficking and migration through tissues is critical for adaptive immune function and, to perform their roles, T cells must be able to navigate through diverse tissue environments that present a range of mechanical challenges. T cells predominantly express two members of the formin family of actin effectors, Formin-like 1 (FMNL1) and mammalian diaphanous-related formin 1 (mDia1). While both FMNL1 and mDia1 have been studied individually, they have not been directly compared to determine functional differences in promoting T cell migration. Through *in vivo* analysis and the use of *in vitro* 2D and 3D model environments, we demonstrate that FMNL1 and mDia1 are both required for effective T cell migration, but they have different localization and roles in T cells, with specific environment-dependent functions. We found that mDia1 promotes general motility in 3D environments in conjunction with Myosin-II activity. We also show that, while mDia1 is almost entirely in the cytoplasmic compartment, a portion of FMNL1 physically associates with the nucleus. Furthermore, FMNL1 localizes to the rear of migrating T cells and contributes to efficient migration by promoting deformation of the rigid T cell nucleus in confined environments. Overall, our data indicates that while FMNL1 and mDia1 have similar mechanisms of actin polymerization, they have distinct roles in promoting T cell migration. This suggests that differential modulation of FMNL1 and mDia1 can be an attractive therapeutic route to fine-tune T cell migration behavior.

## Introduction

T cells require the ability to migrate through diverse environments to patrol for cognate target cells and antigen-presenting cells in peripheral and lymphoid tissues (1–3). This migratory ability involves migration on the surface of, and extravasation through, endothelial layers of blood vessels, as well as navigation of complex, extracellular matrix- (ECM) and/or cell-rich tissues. These migration processes are facilitated by dynamic rearrangement of the actin cytoskeleton in response to environmental and intracellular cues (4–7). While T cell migration and infiltration into tissues is required for controlling infections, it is also necessary for the development and progression of autoimmune disorders, such as Type 1 Diabetes and Multiple Sclerosis, both T cell mediated diseases (8–10). Conversely, solid tumors can generate highly restrictive barriers that significantly limit T cell infiltration and tumor clearance (11–13).

There are several modes of migration that mammalian cells can utilize, including bleb-based motility, mesenchymal crawling, and amoeboid migration (6, 14). The ability of T cells to rapidly switch between amoeboid and adhesion-based mesenchymal modes of migration reflects the need for highly dynamic cytoskeleton mechanics (15). T cell amoeboid migration can happen in 2-dimensional (2D) and 3-dimensional (3D) contexts (16, 17). 2D migration reflects the environment found on the surface of endothelial layers in blood vessel walls and is adhesion-dependent through interactions with integrins and other biomolecules expressed by the endothelium (18). 3D migration can occur in both lymphoid organs that have a high cell density, but relatively low ECM density, and peripheral tissues which have high ECM density (1, 19). While T cell 3D migration in collagen matrices *in vitro* and lymph nodes *in vivo* is thought to be largely integrin-independent, a requirement for integrins in T cell migration through ECM-rich tissues has been identified during local infection responses due to environmental modifications mediated by infection or inflammation (20–22).

The highly dynamic actin cytoskeleton that facilitates these migration modes is regulated by several actin effector protein families (5). One group of actin effectors is the formin family, which is comprised of ~15 proteins in mammals (23). Formins are large, multi-domain proteins that homodimerize via the formin homology 2 (FH2) domain. When dimerized, the formin homology 1 (FH1) domain rapidly binds profilin-bound actin and mediates actin filament growth through processive elongation of either pre-existing filaments or newly nucleated actin dimers and trimers (24). In addition to the FH1 and FH2 domains, most formins possess an N-terminal GTPase binding domain (GBD), a diaphanous inhibitory domain (DID), and a C-terminal diaphanous autoregulatory domain (DAD) (25). When inactive, the formin monomer is kept in a closed conformation by interactions between the distal DID and DAD domains. When the GBD binds an active Rho-GTPase, the autoinhibitory DID-DAD interaction is released and the open conformation formin can homodimerize. Other factors are likely required to fully activate formins, but the DID-DAD interaction represents a primary regulatory mechanism. In addition to the role of formins in polymerizing actin filaments, many formins have also been shown to have microtubule regulation activity. While the formin family consists of many proteins, T cells reportedly highly express 2 out of ~15 family members, formin-like 1 (FMNL1) and mammalian diaphanous-related formin 1 (mDia1) (26–28).

Early investigations of FMNL1 revealed that FMNL1 is overexpressed in T cell lymphomas and ectopic expression in other cancers is associated with aggressive infiltration and poor patient prognosis (29, 30). We have previously demonstrated that FMNL1 is critical for T cell trafficking to sites of inflammation via promotion of migration through restrictive endothelial barriers (31). In that study, we found that FMNL1-deficient T cells had impairments in both trafficking to inflamed tissues and induction of autoimmune disease. In previous literature, mDia1 has been shown to promote T cell migration and adhesion both *in vivo* and *in vitro (* 32–34). Additionally, mDia1-deficiency is associated with impaired thymic egress and lymphopenia (32, 34). These data suggest that FMNL1 and mDia1, individually, are important cytoskeletal effectors with non-redundant functions in T cells.

Many studies investigating formins have focused on a single member of the formin family or have grouped formins together as a single entity, particularly when using the pan-formin inhibitor SMIFH2 (35, 36). This limits the ability to differentiate between the roles of these similar but distinct proteins. There have been efforts to modify SMIFH2 to target it to specific members of the formin family, however chemical modifications to the SMIFH2 scaffold only modulated inhibition strength rather than specificity (37). While the molecular mechanisms of FMNL1- and mDia1-mediated actin polymerization have been described on an individual basis, there has been little work comparing the cellular mechanism by which these formins promote T cell migration. We hypothesize that while these two formins have similar actin polymerization functions, FMNL1 and mDia1 have different roles in promoting T cell migration.

## Results

### Formin isoform expression in T cells

To verify the expression pattern of formins in T cells we performed a preliminary screening of formin isoform expression in naïve and activated T cells. These quantitative RT-PCR data confirmed previous findings (26–28) that FMNL1 and mDia1 are the highest expressed formins in both naïve and activated T cells (**Supplementary Figure 1**). mRNA for other formins was detected but only at much lower levels, particularly in activated T cells, in which mRNA levels for FMNL1 and mDia1 were ~5-fold higher than any other formin tested. Our previous work showed that FMNL1 primarily influences effector T cell trafficking (31) and our screening data suggest that FMNL1 and mDia1 are most highly and differentially expressed in activated T cells. Therefore, for these studies we focused on the roles of FMNL1 and mDia1 specifically in activated T cell motility.

### FMNL1 and mDia1 deficiency results in T cell interstitial migration defects *in vivo*

Our group has previously demonstrated that FMNL1 is required for activated T cell migration through micro-fabricated microchannels *in vitro* and trafficking to tissues in autoimmune disease models *in vivo (* 31). However, the possible role of FMNL1 in T cell interstitial migration within tissues *in vivo* had not been investigated. To characterize how FMNL1 regulates migration within tissues, we analyzed T cell migration parameters of wild-type (WT) and FMNL1-deficient T cells in lymph nodes. Furthermore, to begin comparing the effects of FMNL1- vs. mDia1-deficiency, we also analyzed how lack of mDia1 affected T cell motility in similar *in vivo* environments.

**Figure 1:**
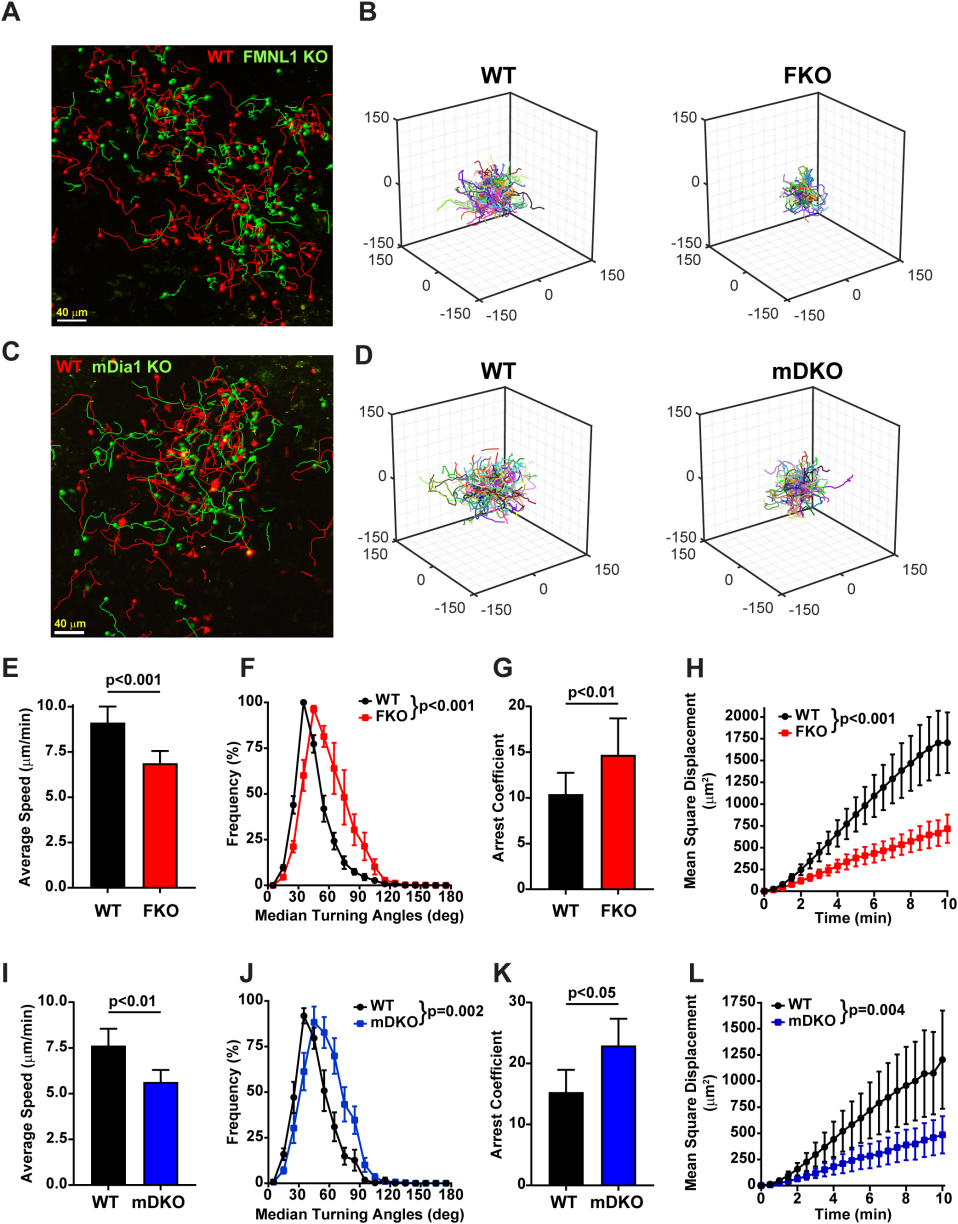
Both FMNL1 and mDia1 regulate T cell motility *in vivo*. WT and FMNL1 KO (FKO) or mDia1 KO (mDKO) T cells were isolated from donor mice, ex vivo activated, differentially dye labeled with VPD or CFSE and co-transferred at a 1:1 ratio into WT recipient mice. Approximately 24 hours later, LNs were harvested and analyzed by time-lapse two-photon microscopy. **(A, C)** Representative maximum Z-projection snapshots from movies showing the migration of WT (red) and FMNL1 KO (green) or WT (red) and mDia1 KO T cells (green). Track lines show the path of T cell movement imaged over 10 minutes. Scale bar = 40 µm. **(B, D)** 3D trajectory plots of WT and FKO or WT and mDKO T cells represented in panels **(A, C)**, respectively. **(E–H)** Quantification of WT and FMNL1 KO T cell average mean track speed, turning angle distribution, arrest coefficient (percentage of each track with instantaneous velocity <2 μm/min), and mean square displacement (MSD) over time. **(I–L)** Quantification of T cell motility parameters of WT and mDia1 KO T cells as in **(E, F)**. Data represent averages ( ± SEM) from a total of 3 experiments. Statistics are paired t tests (for speed and arrest coefficient) and two-way ANOVA (for turning angle and MSD analyses).

We quantified the average speed, turning angle frequency, arrest coefficient (the fraction of time spent moving at less than 2μm/min), and mean squared displacement of WT, FMNL1 KO, and mDia1 KO T cells in lymph nodes (**Figures 1A–D**, and **Supplementary Videos 1**, **2**). We found that FMNL1 deficiency resulted in significant decreases in speed and mean squared displacement, as well as significant increases in median turning angle and mean arrest coefficient (**Figures 1E–H**, **Supplementary Video 1**). Additionally, we confirmed prior findings (33) showing *in vivo* motility defects of mDia1-deficient T cells (**Figures 1I–L**, **Supplementary Video 2**) and identified similar phenotypes between FMNL1 KO and mDia1 KO T cells *in vivo*. These deficiencies suggest reduced efficiency and overall migration capacity of activated T cells lacking FMNL1 or mDia1. Interestingly, deficiency of either formin results in similar phenotypes as described by these metrics. This indicates that even though both formins have similar actin polymerization and microtubule stabilization functions, they are unable to fully compensate for one another, supporting the hypothesis that FMNL1 and mDia1 have non-redundant roles in promoting T cell migration.

### FMNL1 localizes to the rear of migrating T cells while mDia1 distributes evenly in the cytoplasm

**Figure 2:**
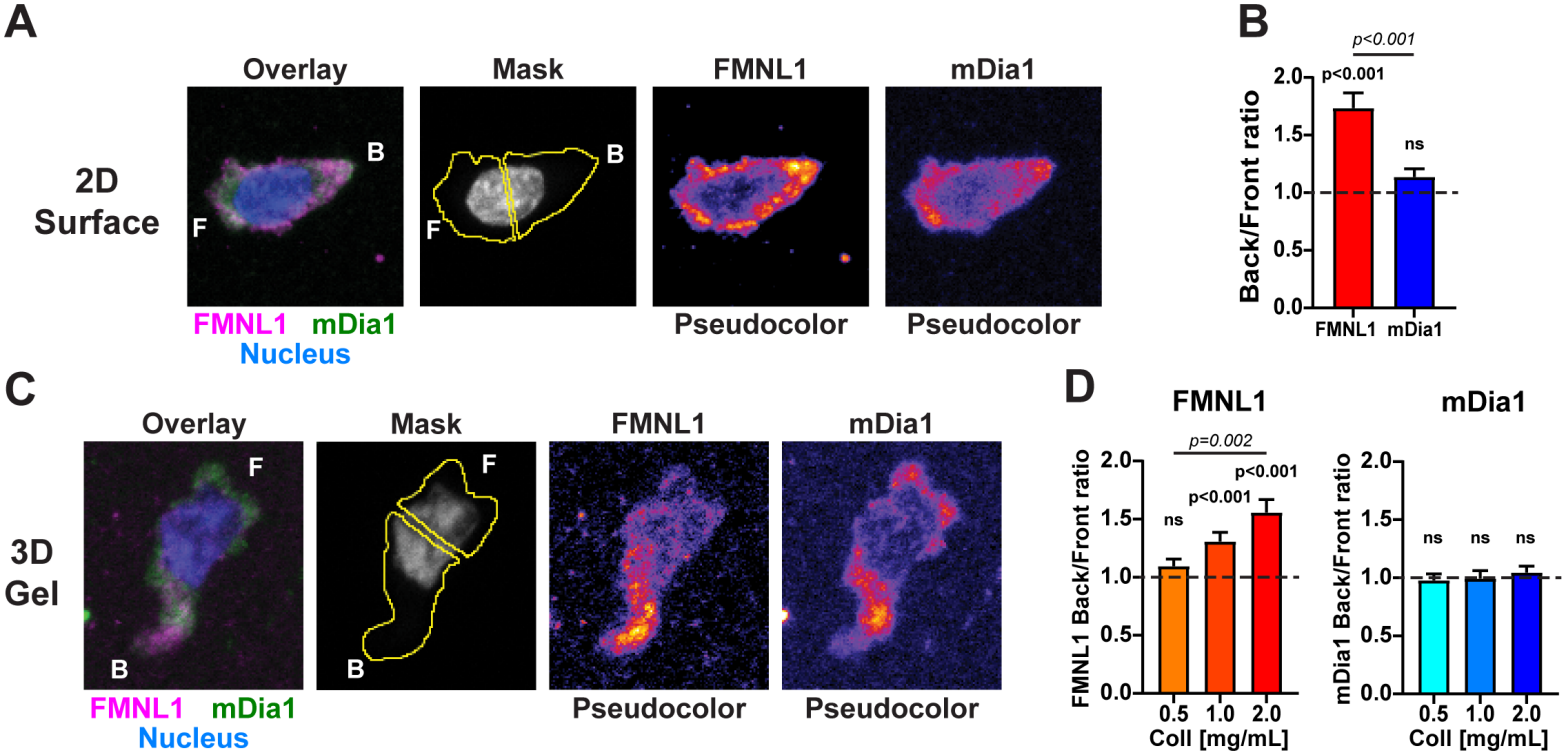
FMNL1 and mDia1 are differentially distributed in migrating T cells. WT T cells were isolated from donor mice, activated ex vivo and then plated on poly-lysine coated slides or embedded in 3D collagen matrices and allowed to migrate for 1-2 hours at 37°C before being fixed and stained. **(A)** Representative sum Z-projection images of fixed and stained activated T cells migrating on 2D poly-lysine coated slides. Left to right: overlay of FMNL1 (purple), mDia1 (green) and nucleus (blue) staining; example of masking scheme for quantifying fluorescence in front and behind the nucleus; and single channel staining for FMNL1 and mDia1 (shown in pseudo-color scale). F indicates the front of the T cell and B indicates the back. **(B)** FMNL1 exhibits posterior enrichment in migrating T cells while mDia1 does not. Quantification of the ratio of back/front fluorescent intensity. Dotted line denotes a ratio of 1 (equal distribution in front and back). **(C)** Representative sum Z-projection images of stained T cells migrating in a collagen matrix, depicted as in **(A)**. **(D)** Left, FMNL1 is enriched in the back of migrating T cells in dense 3D environments (1.0 and 2.0 mg/mL). Right, mDia1 is equally distributed between front and back and this distribution is unaffected by collagen density. Dotted line denotes a ratio of 1 (equal distribution in front and back). Data in **(B)** represents the mean +/- SEM of at least 39 cells pooled from 3 independent experiments. Data in **(D)** are the mean back to front ratio +/- SEM of at least 73 cells pooled from 5 independent experiments. n.s., not significant. For each formin a one-sample t test was done to compare the back/front ratio to the theoretical mean of 1. To compare the distribution between FMNL1 and mDia1 back/front ratios in **(B)** an unpaired t test was performed. To compare between collagen concentrations in **(D)** a one-way ANOVA with Tukey’s post-test was performed. ns, not significant.

Having shown that FMNL1 and mDia1 both promote T cell migration *in vivo*, we next wanted to investigate if and how these two formins may differ in their mechanism of promoting T cell motility. We first examined FMNL1 and mDia1 intracellular localization patterns. Because T cells *in vivo* can migrate on both relatively flat vascular endothelium, as well as three-dimensional cell- or matrix-rich environments, we investigated the intracellular localization of FMNL1 and mDia1 in T cells in both two-dimensional (2D) and three-dimensional (3D) experimental setups. For these experiments, we co-stained fixed T cells for both FMNL1 and mDia1 and based on the distinct polarized morphology of migrating T cells, we measured formin localization as the ratio of back/front distribution of each formin (**Figure 2**).

In T cells crawling on adhesive 2D environments, FMNL1 was significantly localized to the rear portion of the cell whereas mDia1 had no significant polarization between the front and back, indicating a relatively even distribution throughout the T cell (**Figures 2A, B**). Accordingly, FMNL1 had a significantly higher back/front ratio than mDia1 (**Figure 2B**).

For 3D environments, T cells were embedded in collagen matrices of varying concentration, representing T cell migration through low, medium, and high relative densities of ECM. Staining for mDia1 showed no significant back/front bias at any concentration of collagen. Interestingly, FMNL1 had an environmental density-dependent back/front bias with no distribution bias at low collagen density and an increasingly larger rearward bias at intermediate and high collagen concentrations (**Figures 2C, D**).

Taken together, these data suggest that FMNL1 and mDia1 have distinct spatial localization patterns in migrating T cells, with FMNL1 uniquely localizing to the rear of the cell in response to environmental cues while mDia1 remains more evenly distributed throughout the cell. Overall, this further supports the idea that these two formins have distinct roles in T cell migration.

### FMNL1 and mDia1 differentially regulate T cell motility depending on environmental context

Deficiency of either FMNL1 or mDia1 leads to defects in T cell interstitial migration *in vivo* (**Figure 1**). Additionally, we found that FMNL1 and mDia1 have different intracellular localization patterns dependent on the environment (**Figure 2**). Given these data, we wanted to quantify various motility parameters in both 2D and 3D contexts to determine how FMNL1 and mDia1 each contribute to T cell migration in different environments. To determine the context in which each formin is important, we analyzed migration on 2D adhesive surfaces and in 3D collagen matrices of varying densities.

**Figure 3:**
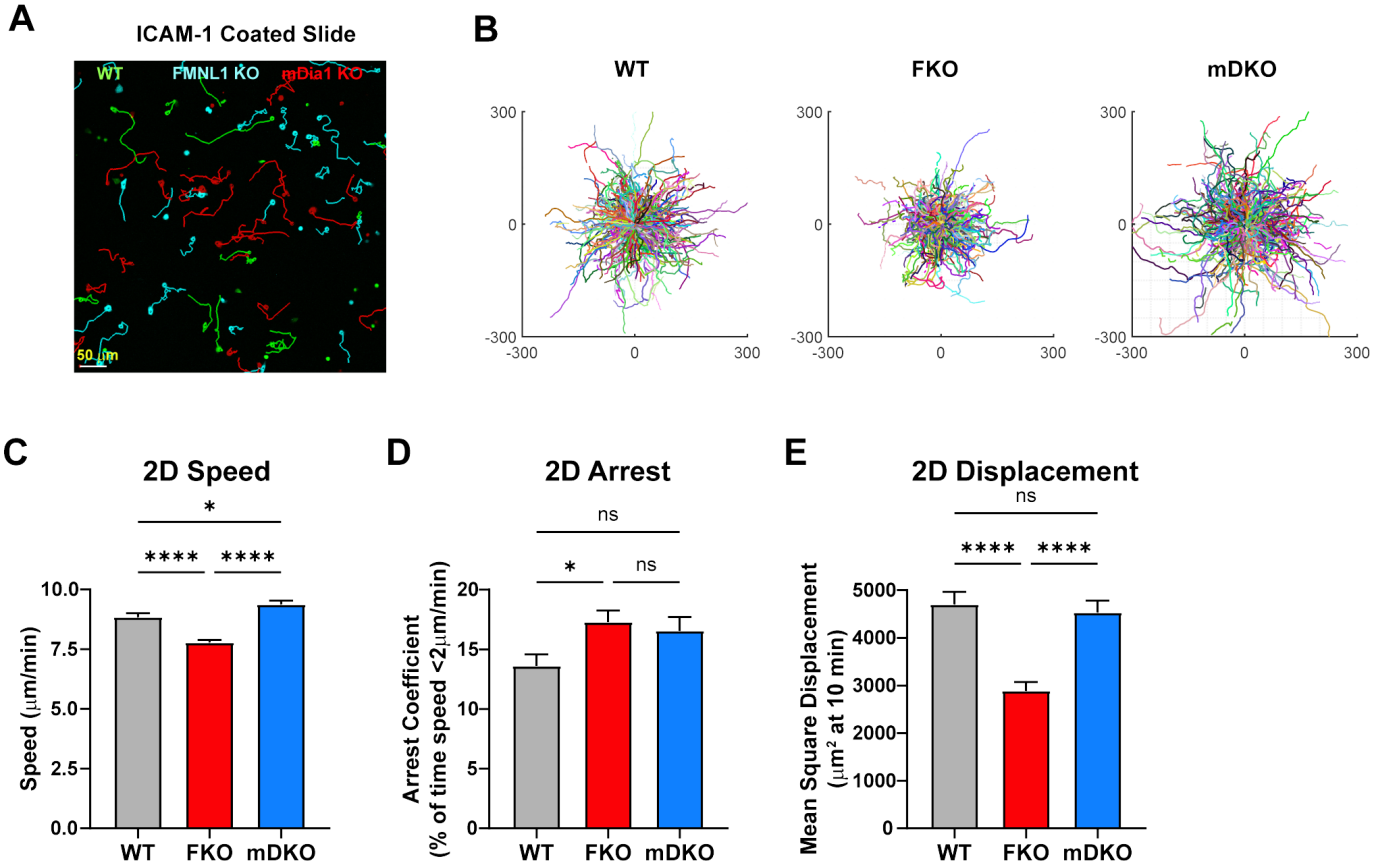
FMNL1 promotes efficient migration on 2D adhesive surfaces. WT, FMNL1 KO, and mDia1 KO T cells were isolated from donor mice, ex vivo activated, and differentially labeled using CFSE, CTV, or CTFR. The cells were mixed and plated on ICAM-1 coated slides and allowed to migrate freely at 37°C for 1-2 hours before imaging. **(A)** Representative sub-region maximum Z-projection snapshot showing migration of WT (green), FMNL1 KO (cyan), and mDia1 KO (red) T cells migrating on an ICAM-1 coated surface. Track lines show the T cell path over 30 minutes. **(B)** Trajectory plots of WT, FKO, and mDKO T cells migrating on ICAM-1 from the complete data set represented by the sub-region in panel **(A)**. **(C–E)** 2D quantifications of average track speed **(C)**, average arrest coefficient (% timepoints with instantaneous speed below 2 μm/min) **(D)**, and mean squared displacement of cells tracked continuously for 10 minutes **(E)**. Data in **(C–E)** represents the mean +/- SEM of at least 450 cells pooled from 3 independent experiments. Significance was determined by Brown-Forsythe and Welch ANOVA tests with Games-Howell’s multiple comparisons test. ns, not significant, * = p<0.05, **** = p<0.0001.

Using ICAM-1 coated 2D surfaces, to model the surface of an endothelial layer, we found that FMNL1 KO T cells had significantly decreased average speed and mean squared displacement, and an increase in arrest coefficient (**Figures 3A–E**, **Supplementary Video 3**). On the other hand, mDia1 KO T cells had no significant impairment in the motility parameters analyzed relative to WT T cells. This was surprising as mDia1 has previously been shown to promote migration in different 2D contexts (33), and we expected FMNL1-deficient T cells to not have migration defects in the absence of environmental restrictions. This raises the possibility that FMNL1 may also be important for T cell detachment from adhesive surfaces, while mDia1 may have roles dependent on additional environmental cues.

**Figure 4:**
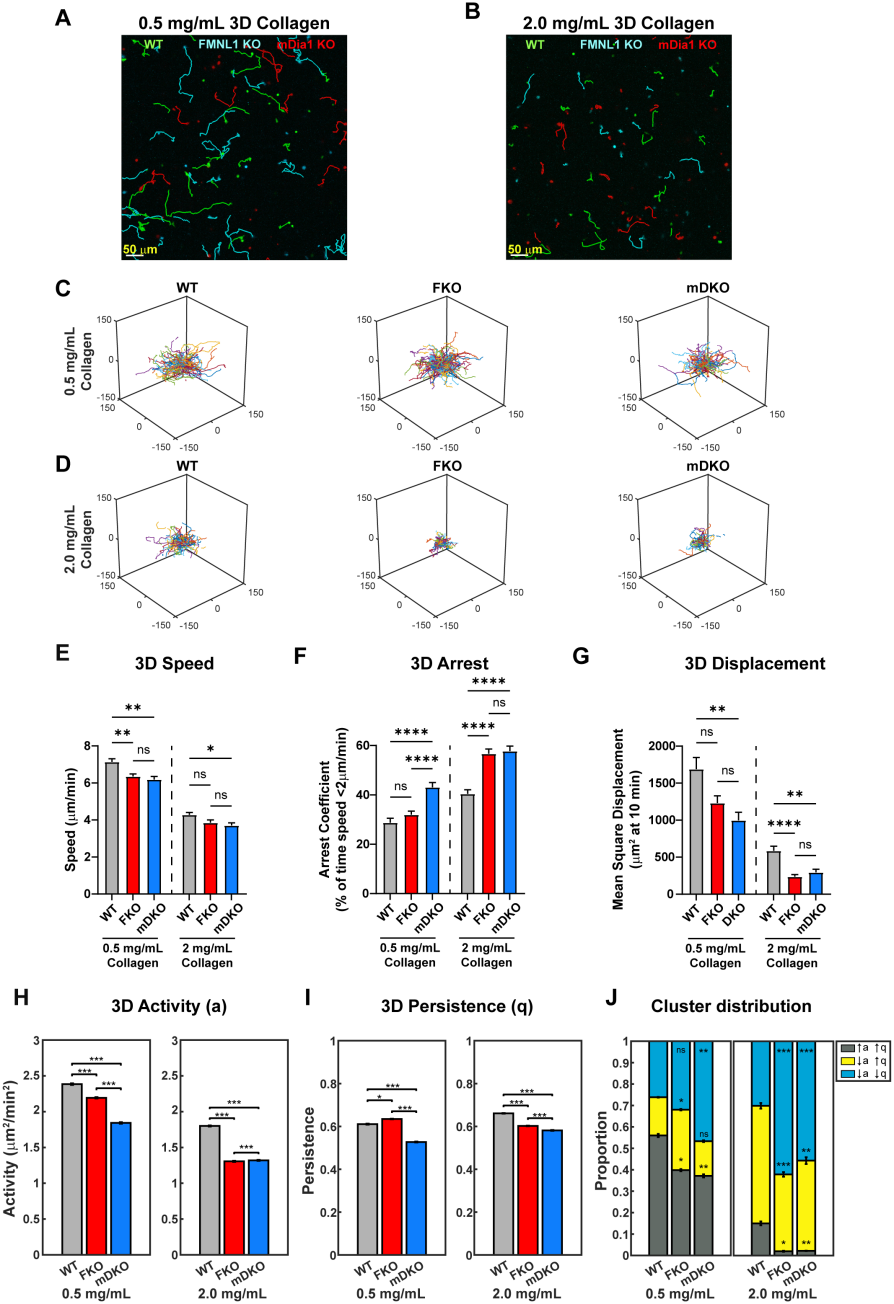
The roles of FMNL1 and mDia1 in T cell migration are dependent on environmental context. WT, FMNL1 KO, and mDia1 KO T cells were isolated from donor mice, ex vivo activated, and differentially labeled using CFSE, CTV, or CTFR. The cells were embedded in collagen matrices and allowed to migrate at 37°C for 1-2 hours before imaging. **(A, B)** Representative maximum Z-projection showing migration of WT (green), FMNL1 KO (cyan), and mDia1 KO (red) T cells in 0.5 mg/mL **(A)** or 2.0 mg/mL **(B)** collagen matrices. Track lines show the T cell path over 30 minutes. **(C, D)** Trajectory plots of WT, mDKO, and FKO T cells migrating in 0.5 mg/mL **(C)** or 2.0 mg/mL **(D)** collagen matrices. **(E–G)** 3D quantifications of average track speed **(E)**, average arrest coefficient **(F)**, and mean squared displacement of cells tracked continuously for 10 min **(G)**. **(H, I)** Average activity **(H)** and persistence **(I)** calculated from persistent random walk analysis. **(J)** Stacked bar plots representing the proportion of cells clustering to three motility modes [high activity and persistence (↑a↑q), low activity and high persistence (↓a↑q), and low activity and persistence (↓a↓q)] in 0.5 and 2.0 mg/mL collagen matrices. Data in **(E–G)** represents the mean +/- SEM of at least 70 cells per condition pooled from 4 independent experiments. Data in **(H-I)** represent the mean +/- SEM of all instantaneous velocity vectors of at least 70 cells per condition pooled from 4 independent experiments. Data in **(J)** represents the mean +/- SEM of n=100 bootstrap resampling repetitions. Significance in **(E–G)** was determined by Brown-Forsythe and Welch ANOVA tests with Games-Howell’s multiple comparisons test. Significance in **(H-I)** was determined by Mann-Whitney U test. Significance in **(J)** between WT and FKO or mDKO groups was determined by a two-population proportion Z test. ns, not significant, * = p<0.05, ** = p<0.01, *** = p<0.001, **** = p<0.0001.

We next analyzed motility in 3D environments by embedding T cells in collagen matrices of various densities (**Figure 4**, **Supplementary Figure 2**, and **Supplementary Videos 4**, **5**). Relative to WT T cells, mDia1 KO T cells had significant defects in all three parameters analyzed (speed, arrest coefficient, and mean square displacement) regardless of the collagen density (**Figures 4E–G**). Interestingly, however, the phenotype of FMNL1 KO T cells was more dependent on the environment density. In low collagen density matrices, FMNL1 KO T cells only had a small reduction in speed, but no significant defects in arrest and displacement. Conversely, in high density 3D environments, FMNL1 KO T cells had significant defects in both arrest coefficient and mean squared displacement (**Figures 4E–G**). These data support that in 3D environments FMNL1 is needed for efficient T cell migration through restrictive environments, whereas mDia1 is important for 3D motility in general.

To further quantify the T cell motility phenotype in 3D environments, we analyzed T cell activity (a) and persistence (q) by fitting the cell trajectories to a persistent random walk model (38). We compared the activity and persistence of the T cell populations in both the low- and high-density collagen (**Figures 4H, I**). We found that in the low-density collagen matrices, both FMNL1- and mDia1-deficiency led to significantly lower activity compared to WT, with FMNL1-deficient cells having an intermediate activity (**Figure 4H**). However, in high-density collagen, FMNL1- and mDia1-deficient cell activity is significantly lower than WT and nearly equivalent to each other. Interestingly, when comparing persistence, FMNL1-deficiency was associated with a small, but significant increase in low-density environments, but a significant decrease in high-density environments relative to WT T cells (**Figure 4I**). In contrast, mDia1-deficient T cells had a significant decrease of migratory persistence in both low- and high-density collagen matrices. The improved persistence of FMNL1 KO T cells in low-density collagen likely compensated these cells’ reduced speed to prevent a significant drop of the mean squared displacements as compared with WT cells, explaining the phenotypic dependence of FMNL1-deficient cells on collagen density.

Next, we investigated the heterogeneity of the activity and persistence parameters within T cell populations to identify novel phenotypes in sub-populations of cells that may be obscured by whole population averages of motility parameters. Thus, we performed k-means clustering based on each cell’s activity and persistence values, identifying three sub-populations of T cells: high activity and persistence (↑a↑q), low activity and high persistence (↓a↑q), and low activity and persistence (↓a↓q) (**Supplementary Figure 2A**). We then compared the proportions of these three sub-populations in low- and high-density collagen for WT, FMNL1 KO, and mDia1 KO T cells (**Figure 4J**, **Supplementary Figures 2B–G**). In low-density collagen, deficiency of FMNL1 or mDia1 are both associated with significant reductions of the high motility (↑a↑q) sub-population. This reduction was partially balanced by an increase of the ↓a↑q sub-population in FMNL1-deficient T cells, but mDia1-deficient cells had a large increase in the ↓a↓q population (as shown by the shifts in probability contours of a and q in **Supplementary Figures 2B–D**). In high-density collagen, deficiency of either formin led to the ↑a↑q sub-population being virtually eliminated with significant increases to the size of the ↓a↓q sub-population (**Figure 4J**).

Taken together, these data indicate that deficiency of either FMNL1 or mDia1 is associated with different phenotypes depending on environmental context. Furthermore, the difference in cluster proportions in low density collagen supports the hypothesis that FMNL1 and mDia1 have different mechanisms for promoting motility, specifically in modulating the persistence of migration in low density environments.

### FMNL1 promotes T cell migration through a Myosin-II independent pathway

**Figure 5:**
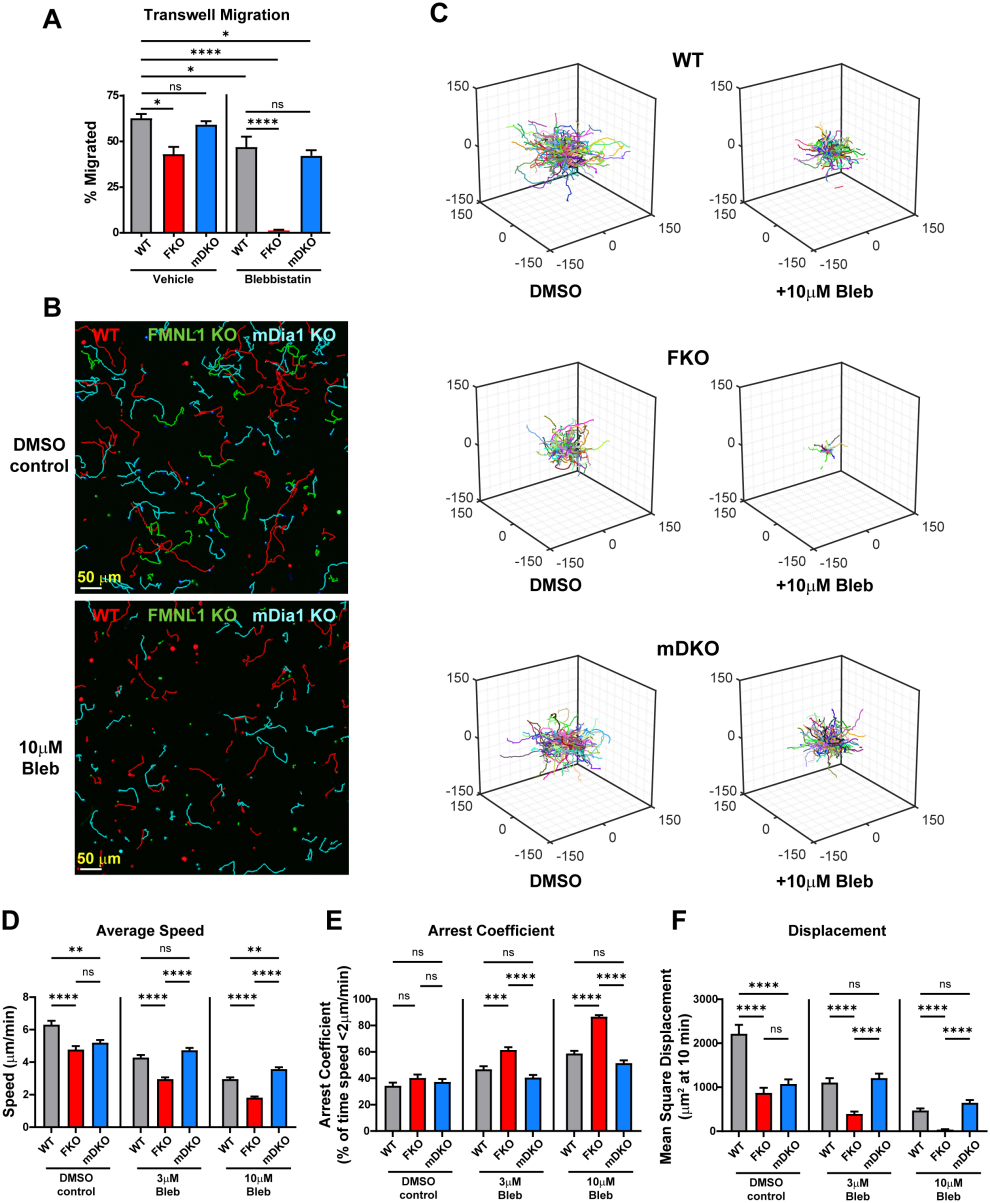
FMNL1 mediates T cell migration independently of Myosin-II. WT, FMNL1 KO, and mDia1 KO T cells were collected from donor mice, ex vivo activated, and differentially fluorescently labeled. **(A)** T cells were seeded onto 3μm transwell inserts with 50μM Blebbistatin or a vehicle control and 100 ng/mL CXCL10 in the bottom well. Percent migration is calculated as the number of cells counted in the bottom well normalized to a 20% loading control well. **(B–F)** T cells were embedded in 1.0 mg/mL collagen with increasing concentrations of para-nitro-Blebbistatin. **(B)** Representative maximum Z-projection images of WT (red) FMNL1 KO (green), and mDia1 KO (cyan) T cell migration through 1.0 mg/mL collagen matrices treated with either DMSO (top) or 10 μM para-nitro-Blebbistatin (bottom). Track lines show the path taken by the cells over 25 min. **(C)** Trajectory plots of the DMSO and 10 μM Blebbistatin-treated groups for all cells analyzed in **(D–F)**. **(D–F)** Quantification of mean track speed, average arrest coefficient, and mean squared displacement of cells tracked continuously for 10 min. Cells were treated with a DMSO control, 3 μM or 10 μM para-nitro-Blebbistatin. Data in **(A)** represents the mean +/- SEM of three independent experiments. Significance was determined by One-way ANOVA. Data in **(D–F)** represent the mean +/- SEM of at least 165 cells per condition pooled from three independent experiments. Significance was determined by Brown-Forsythe and Welch ANOVA tests with Games-Howell’s multiple comparisons test. ns, not significant, * = p<0.05, ** = p<0.01, *** = p<0.001, **** = p<0.0001.

Our data so far has established that FMNL1 and mDia1 are both required for T cell migration *in vivo* and in 3D environments *in vitro*, and we have identified distinct localization patterns and different impacts on T cell migration of these two formins in an environment-dependent manner. To further elucidate the differences between FMNL1 and mDia1 in regulating T cell migration, we next wanted to investigate the effector pathways that they act through. Through their actin polymerization activity, formins generate actin filaments that can promote motility independently or in concert with Myosin-II. Thus, to assess if Myosin-II contributed to FMNL1- or mDia1-mediated T cell migration, we first performed transwell chemotaxis assays using restrictive 3 µm pores (**Figure 5A**). To interrogate the role of Myosin-II in FMNL1- and mDia1-mediated T cell migration, we inhibited Myosin-II activity with Blebbistatin (39). In this setting, both Myosin-II inhibition and FMNL1-deficiency resulted in significantly reduced T cell migration compared to vehicle treated WT T cells, while mDia1-deficient T cell migration was not significantly different than WT controls. Furthermore, mDia1-deficient T cells treated with Blebbistatin did not show an additive or synergistic reduction in T cell migration, suggesting they may function through the same pathway. In contrast, Myosin-II inhibition combined with FMNL1-deficiency reduced T cell migration to nearly zero (**Figure 5A**), showing synergy between FMNL1 deficiency and Myosin-II inhibition and indicating non-redundant effects of these two cytoskeletal effectors.

Since FMNL1 can have an environment-density dependent impact, we then wanted to also evaluate the interaction of FMNL1 and Myosin-II pathways in a 3D context. To complement our transwell chemotaxis assay, we thus analyzed T cell motility in 3D collagen matrices in the presence or absence of para-nitro-Blebbistatin, a derivative of Blebbistatin that has reduced phototoxic effects in cells (40) (**Figure 5**, **Supplementary Figure 3**, and **Supplementary Videos 6**, **7**). Here, we used an intermediate collagen density of 1 mg/mL to ensure that the effect of environmental density on motility would not overshadow the effect of Myosin-II inhibition. We found that para-nitro-Blebbistatin treatment led to dose-dependent defects in T cell motility parameters (speed, arrest, and displacement). In the absence of Myosin-II inhibition in these 3D collagen matrix conditions, the motility defects of FMNL1- and mDia1-deficient T cells were similar with significant impairment in both speed and displacement (**Figures 5D–F**, DMSO control group, **Supplementary Video 6**). Interestingly, upon Myosin-II inhibition, the phenotype of FMNL1 KO and mDia1 KO T cells diverged. The motility of FMNL1 KO T cells became progressively more impaired with Myosin-II inhibition; in contrast the motility phenotype of WT and mDia1 KO T cells became equivalent (**Figures 5D–F** 3μM and 10μM Blebbistatin groups, **Supplementary Video 7**). The exception was cell speed at 10μM Blebbistatin, where mDia1 KO T cells were slightly faster than WT cells. Additionally, Myosin-II inhibition (at 10μM Blebbistatin) and FMNL1-deficiency had additive effects, with T cell motility almost completely abrogated in these conditions (**Figures 5C, F**).

Similar to **Figure 4**, we also performed k-means clustering based on the activity and persistence values of WT and formin KO T cells with or without Blebbistatin treatment (**Supplementary Figure 3A**). Myosin inhibition in WT T cells resulted in a general shift towards lower activity with a large enrichment of the medium motility cluster (the ↓a↑q sub-population) and, to a lesser extent, also an increase in the low motility cluster (↓a↓q sub-population). In the FMNL1 KO T cells Blebbistatin treatment caused an almost complete shift to low motility cluster. Conversely, in the mDia1 KO T cells, similarly to WT cells, Blebbistatin treatment resulted in an increase of the medium motility cluster only with no significant difference in the low motility cluster, indicating an activity change without affecting persistence.

Overall, this further confirms our transwell findings on the interaction between FMNL1-deficiency and Myosin-II inhibition, which suggests that FMNL1 and Myosin-II act through different pathways.

### mDia1 is needed for the T cell motility enhancement mediated by microtubule destabilization in restrictive environments

While formins primarily act by polymerizing actin, both FMNL1 and mDia1 can also have roles in regulating microtubules (28, 33, 41). Furthermore, a recent publication demonstrated that microtubule destabilization via nocodazole treatment improves motility of primary T cells in collagen hydrogels (42). The release of the Rho guanine exchange factor (GEF)-H1 from destabilized microtubules has been previously shown to activate RhoA, which can then activate Myosin-II downstream of ROCK and thus enhance motility (43–45). Interestingly, RhoA is also reported to be an activator of mDia1 (46, 47).

**Figure 6:**
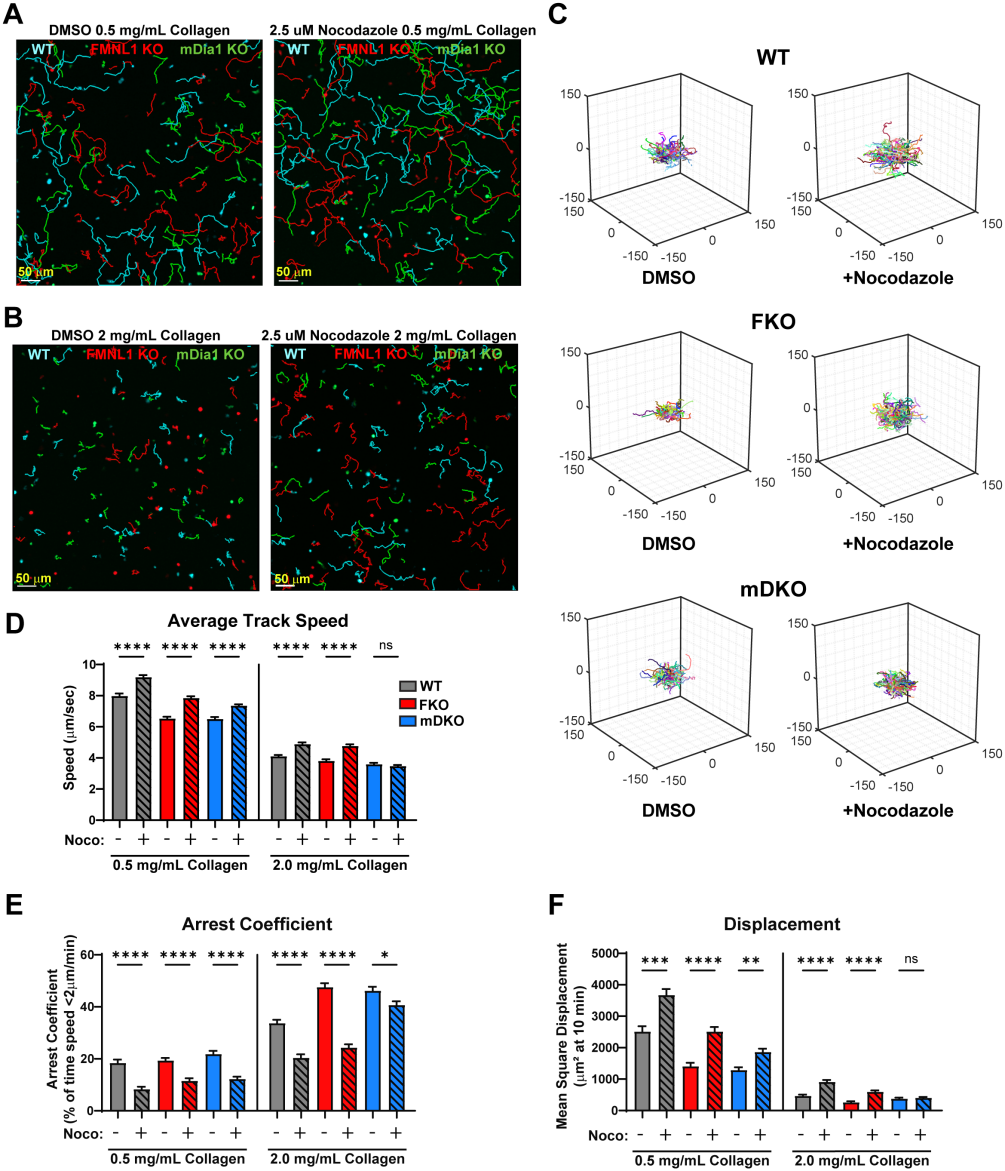
Nocodazole enhances T cell migration parameters, except for mDia1 KO T cells in high density environments. WT, FMNL1 KO, or mDia1 KO T cells were collected from donor mice, ex vivo activated, then differentially fluorescently labeled and embedded in collagen matrices with either a DMSO control or 2.5 μM nocodazole. **(A, B)** Representative maximum Z-projection images of T cell migration in 0.5 mg/mL **(A)** or 2.0 mg/mL **(B)** collagen in the presence of DMSO (left) or 2.5 μM nocodazole (right). Track lines show the path taken by T cells over 25 min. **(C)** Trajectory plots of DMSO and nocodazole-treated T cells in 2.0 mg/mL collagen for all cells analyzed in **(D–F)**. **(D–F)** Quantification of mean track speed, average arrest coefficient, and mean squared displacement of cells tracked continuously for 10 min. Data represents the mean +/- SEM of at least 96 cells per condition pooled from 3 independent experiments. Significance was determined by Brown-Forsythe and Welch ANOVA tests with Games-Howell’s multiple comparisons test. ns, not significant, * = p<0.05, ** = p<0.01, *** = p<0.001, **** = p<0.0001.

To further interrogate the pathways by which FMNL1 and mDia1 promote T cell migration, we investigated the functional interaction between these formins and microtubule network destabilization. We first established the ideal dosage to minimize potential toxicity and off-target effects of nocodazole and found that 2.5µM nocodazole produced the largest effective motility enhancement (**Supplementary Figure 4**). We then analyzed the migration-enhancing effect of nocodazole on WT and formin KO T cells in low- and high-density collagen matrices (**Figure 6**, **Supplementary Figure 5**, and **Supplementary Videos 8**-**11**). We quantified T cell motility parameters in WT and each formin KO T cells with and without microtubule destabilization (**Figures 6A–C**). In low-density collagen matrices, nocodazole treatment significantly improved T cell speed, arrest, and displacement independent of formin genotype (**Figures 6D–F**, 0.5mg/mL collagen groups, **Supplementary Videos 8**, **9**). However, in high-density 3D collagen matrices, nocodazole only improved migration in WT and FMNL1 KO T cells. In contrast, mDia1 KO T cells treated with nocodazole in high density environments showed no significant improvement in speed and displacement, and only a minor reduction in the arrest coefficient (**Figures 6D–F**, 2mg/mL collagen groups, **Supplementary Videos 10**, **11**).

We also performed k-means clustering of WT and formin KO T cells with or without Nocodazole treatment in 2 mg/mL collagen (**Supplementary Figure 5A**). This analysis showed that in WT T cells Nocodazole treatment resulted in an upward shift in the clusters from low motility (the ↓a↓q sub-population) to medium motility (↓a↑q sub-population) and from medium motility to high motility (↑a↑q sub-population). In the FMNL1 KO T cells there was a similar upward shift, mainly from the low motility cluster to the medium motility cluster, and an increase in the high motility population as well. However, in the mDia1 KO T cells there was an enrichment of the medium motility cluster but no increase in the high motility cluster, indicating an increase in persistence only but not in activity.

Overall, this suggests that the increased T cell migration following destabilization of microtubules relies in part on mDia1 in high-density environments but is independent of FMNL1.

### FMNL1 promotes T cell motility by mediating nuclear deformation in restrictive environments

We have previously shown that WT T cells rapidly polymerize actin directly behind the nucleus when migrating through restrictive openings in microchannels (31). In FMNL1-deficient T cells, this actin polymerization is mostly absent, and T cells are impaired in entering and traversing the microchannel constriction point (31). Based on these previous data, we investigated nuclear deformation in migrating T cells in 3D environments and the role of formins in mediating this nucleus deformation. We thus quantified the nuclear ellipticity of migrating T cells embedded in microsphere-labeled 3D collagen over time.

**Figure 7:**
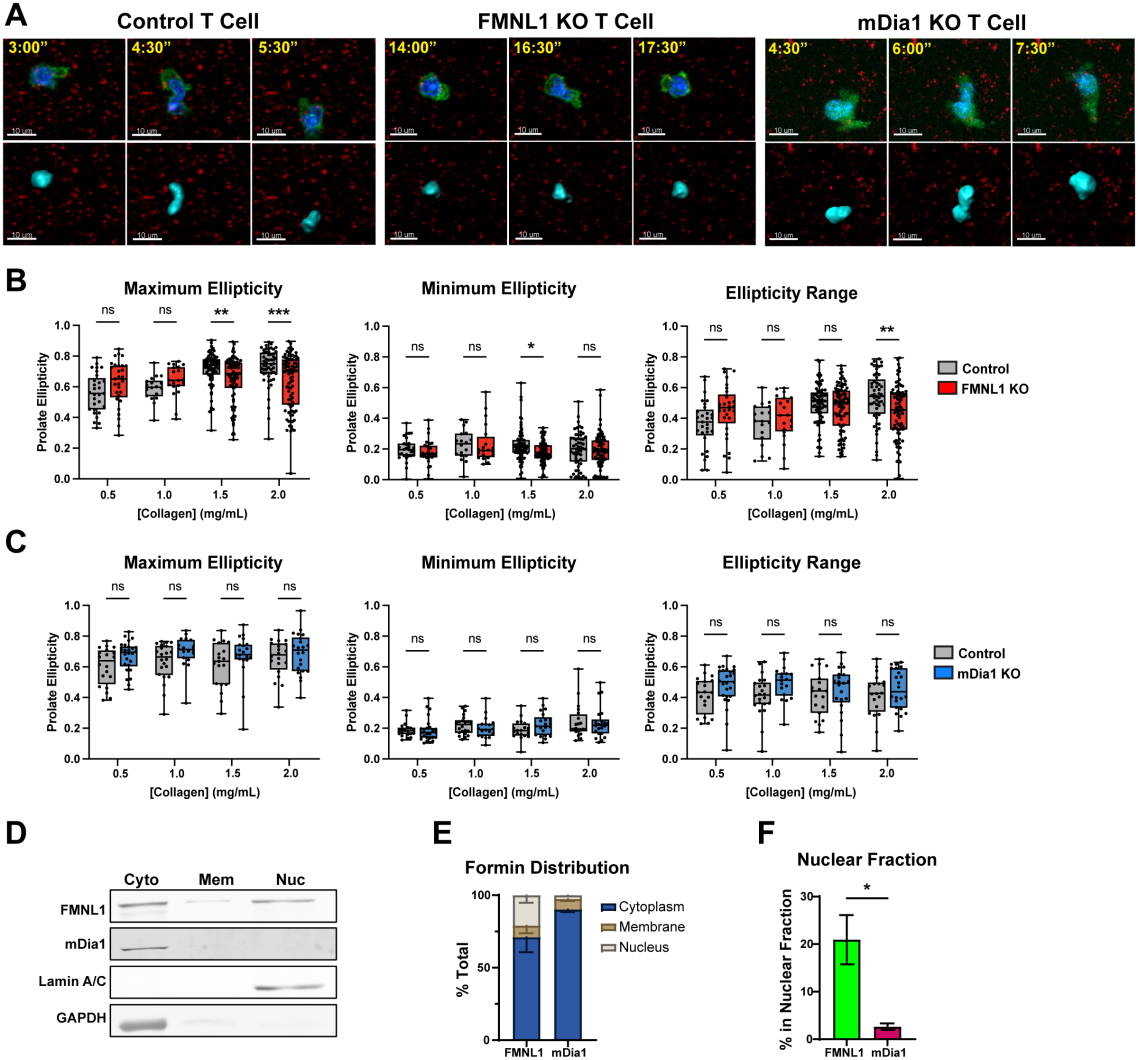
FMNL1 mediates nuclear deformation in restrictive environments via interactions with the nucleus. **(A–C)** Control, FMNL1 KO, or mDia1 KO T cells were collected from LifeAct-GFP positive or negative donor mice and ex vivo activated. The cells were stained with Hoechst nuclear dye and embedded separately in collagen matrices seeded with fluorescent microspheres. The T cell cortex and cytoplasm was visualized with LifeAct-GFP or an additional CFSE dye labeling step. **(A)** Representative images of control, FMNL1 KO, and mDia1 KO T cells before, during, and after engaging a restriction in 2.0 mg/mL collagen. Top: Green = LifeAct-GFP or CFSE, Blue = Nucleus, Red = 0.2 μm microspheres. Bottom: Corresponding software rendering of the nuclear surface (cyan) and red microspheres. Time is represented as min:sec. **(B, C)** Maximum, minimum, and range (max – min) of prolate ellipticity for control and FMNL1 KO **(B)** or control and mDia1 KO **(C)** T cells. **(D–F)** WT T cells were collected from donor mice, ex vivo activated and lysed using a serial subcellular fractionation kit to collect cytoplasmic, membrane, and the non-chromatin bound nuclear compartments. **(D)** Representative Western blot of subcellular fractionation experiments showing the three analyzed fractions. Lamin A/C and GAPDH were probed as controls for the nucleus and cytoplasm compartments respectively. **(E)** Densitometry quantification of the percent of total FMNL1 and mDia1 present in each compartment, normalized to the amount of total lysate used for the experiment. **(F)** Comparison of the percentage of total FMNL1 and mDia1 present in the nuclear fraction alone. Data in **(B, C)** represent the average ellipticity values +/- SEM for at least 18 cells per condition pooled from 6 [in **(B)**] or 4 [in **(C)**] independent experiments. Data in **(E, F)** represent formin percentages +/- SEM averaged from 4 independent experiments. Significance in **(B, C)** was determined by Two-way ANOVA with Holm-Sidak’s multiple comparisons test. Significance in **(F)** was determined by an unpaired t-test. ns, not significant, * = p<0.05, ** = p<0.01, *** = p<0.001.

To evaluate the utility of prolate ellipticity as a measure of nuclear dynamics, we first compared the average maximum and minimum ellipticity values of WT T cells migrating over time in 3D collagen gels from representative data sets and found that the maximum ellipticity was significantly higher than the minimum (**Supplementary Figure 6A**), supporting that there are significant oscillations in nucleus shape during T cell migration. We also examined nuclear ellipticity values for control T cells before, during, and after traversing a restriction marked by fluorescent microspheres. We found that control T cells have a large dynamic range of values with the maximum ellipticity typically occurring at the frame when the T cell is actively engaged with a restriction (**Figure 7A** control T cell and **Supplementary Figure 6B**). Together these quantifications demonstrate that prolate ellipticity is a suitable metric for examining the relationship between formins and nuclear deformation during confined 3D T cell migration.

We embedded control, FMNL1 KO, or mDia1 KO T cells in a range of collagen matrix densities and obtained the maximum and minimum nucleus ellipticity values, and the ellipticity range (maximum ellipticity minus minimum ellipticity) for individual T cells (**Figures 7A–C**). For T cells in 0.5 and 1.0 mg/mL collagen matrices (low-intermediate density), control T cell nuclear deformation was not significantly different from either FMNL1 KO or mDia1 KO T cells. However, in 1.5 and 2.0 mg/mL collagen matrices (intermediate-high density), the maximum nuclear deformation for FMNL1 KO T cells was significantly lower than the control T cells (**Figure 7B**). The minimum ellipticity values were not significantly different except for 1.5 mg/mL, where FMNL1 KO T cells had a slightly lower minimum. Furthermore, analysis of the ellipticity range distribution showed a significant reduction in the range of nuclear deformation in FMNL1 KO T cells in 2.0 mg/mL collagen gels (**Figure 7B**). Conversely, mDia1 KO T cell nuclear deformation was not significantly different from control T cells in any condition (**Figure 7C**). These results, coupled with the data demonstrating a migration defect in high density collagen for FMNL1 KO T cells support that these cells are impaired in deforming the nucleus to efficiently move through restrictions in the environment. This indicates that FMNL1, but not mDia1, is necessary for promoting nuclear deformation through high density, restrictive, 3D matrices.

Given our finding that FMNL1 is important for promoting nuclear deformation, we investigated if FMNL1 was associated with the nuclear compartment. Thus, we performed serial subcellular fractionations on WT T cells to separate the cytoplasmic, membrane, and soluble nuclear (non-chromatin bound) compartments (**Figure 7D**). We then quantified the percentage of total FMNL1 or mDia1 present in each fraction. Here we found that FMNL1 is present in both cytoplasmic and nuclear fractions, whereas mDia1 is localized almost entirely in the cytoplasm (**Figure 7E**). Accordingly, we found that the percentage of FMNL1 associated with the nuclear compartment was significantly higher than that of mDia1 (**Figure 7F**).

These data taken together support that FMNL1 promotes T cell motility in confined environments by mediating nuclear deformation via association with the nuclear compartment (see proposed model in **Supplementary Figure 7**).

## Discussion

The ability for T cells to efficiently migrate through a variety of tissues and directly interact with antigen-presenting cells and target cells is critical to their function. Individual T cells will likely encounter multiple environmental conditions ranging from 2D endothelial surfaces during extravasation from vasculature to wide ranges of 3D environmental compositions when migrating within tissues. This requires a highly dynamic cytoskeletal network to rapidly adapt to these conditions. A family of proteins that elicit dynamic cytoskeletal remodeling is the formin family of actin effectors. Our group has previously established the importance of FMNL1 in T cell extravasation in inflammatory conditions (31). In addition to FMNL1, T cells also express mDia1 and, at much lower levels, mDia3 and several additional formins (48). Previous literature regarding formins in T cells has typically focused on either FMNL1 or the mDia formins individually. The mDia-related publications have consistently demonstrated defects in migration in a variety of contexts for mDia1-deficient T cells and FMNL1-related publications have identified multiple roles for FMNL1 from promoting migration and infiltration to maintaining the structure of the Golgi complex (31–34, 49). However, the distinct mechanisms of action of FMNL1 versus mDia1 in T cell motility in complex environments have not been fully elucidated.

Subcellular localization of proteins is a major factor in defining their function, as localization influences the availability of binding partners and limits activity to defined regions in the cell (50). By demonstrating that FMNL1 and mDia1 have different localization patterns within T cells by confocal microscopy and subcellular fractionation, we provide important evidence supporting that even though FMNL1 and mDia1 have similar actin polymerization activity, they have distinct roles in promoting T cell migration. Furthermore, we found that FMNL1 can also be enriched at the back of T cells migrating on 2D adhesive surfaces. This suggests that while environmental density and complexity are important for the spatial distribution of FMNL1, there is likely another mechanism that is important for its localization independent of the environmental density. It’s important to note that the actin cytoskeleton is inherently dynamic, so experiments on fixed cells and cell fractionations only provide snapshots of cellular activity. To gain a better understanding of the dynamics of formin localization and activation, future experiments using fluorescently tagged formins in live migrating T cells would be of interest.

When we quantified key T cell motility parameters, we found that the specific defects of FMNL1 or mDia1 deficiency were highly dependent on the environment. The *in vivo* migration experiments demonstrated that either FMNL1- or mDia1-deficiency resulted in migration defects, consistent with previous literature on mDia1 (33). However, in our 2D migration experiments, we found that despite the lack of restrictions, FMNL1 KO T cells had significant defects in migration whereas mDia1 KO T cells were largely unaffected. This was surprising based on our previous study on FMNL1 showing that it’s required for migration through restrictions as well as other literature studying mDia1 which shows that it promotes motility on 2D surfaces (31, 33). Since FMNL1 localizes to the rear of the T cell when there is no restriction or 3D matrix, it may also be important for generating forces for detachment of the uropod from the migration surface, explaining the defect on 2D adhesive surfaces. For the 2D migration experiments, we coated the slides with ICAM-1, an integrin which helps T cells firmly adhere to endothelial surfaces (51). The previous study showing that mDia1 promotes 2D motility also used ICAM-1 coated surfaces, but used media containing EGTA and high Mg^2+^ concentration, a cation that strongly activates LFA-1, the adhesion receptor for ICAM-1 (33, 52). Since our media did not have EGTA or additional Mg^2+^ supplementation, it’s possible that the difference between previous data and our findings is due to differences in experimental conditions that influence the activity of integrin interactions. This raises the further possibility that mDia1 may have additional roles in regulating T cell adhesion when integrins are in an active state. In future studies, it will be interesting to investigate the interplay between surface interactions and formin activity to interrogate the role of formins more specifically in 2D contexts.

Our analysis of T cell migration in 3D collagen matrices demonstrated that FMNL1- and mDia1-deficiency have different impacts dependent on the restrictiveness of the environment. This is emphasized by the significant fraction of FMNL1-deficient T cells that can move with high directional persistence through low-density collagen, albeit at a reduced speed. On the other hand, mDia1-deficient T cells experience a loss of both persistence and activity in low-density collagen. Interestingly, in high density collagen both FMNL1- and mDia1-deficient T cells showed a very large increase in the fraction of cells with reduced activity and persistence. These data further reinforce our conclusion that FMNL1 is especially important for promoting migration through highly restrictive environments.

Actin polymerization represents a major component of cell migration. Formins are a major family of actin polymerizing proteins, however formins also have a role in microtubule regulation (41, 53, 54). Additionally, formin functions are known to have crosstalk with Myosin-II, a motor protein that generates contractile force by cross-linking actin filaments (55). In this study, we wanted to determine the cytoskeletal pathways associated with FMNL1 and mDia1 function in the context of migration. We thus investigated if formins interacted with a Myosin-II dependent pathway in T cell migration. We first used transwell assays with 3 μm pore inserts, which is smaller than the average T cell nucleus diameter, requiring active force generation to deform the rigid nucleus. In the presence of Myosin-II inhibition, WT and mDia1 KO T cells showed a small decrease in migration, whereas Blebbistatin-treated FMNL1 KO T cells had nearly no migration capacity compared to control T cells. Further experiments with Blebbistatin in 3D environments showed a dose-dependent migration defect in FMNL1 KO T cells, but mDia1 KO T cells were relatively insensitive to Myosin-II inhibition. Overall, this combinatorial effect between FMNL1 deficiency and Myosin-II inhibition suggests that FMNL1 and Myosin-II act on different cytoskeletal pathways while mDia1 interacts more directly with a Myosin-II pathway to promote T cell motility.

A recent publication showed that microtubule destabilization by nocodazole treatment paradoxically enhanced primary T cell migration in collagen matrices (42). The authors suggested a potential mechanism for this effect where microtubule destabilization releases GEF-H1, activating a RhoA-ROCK-MLC pathway, and leading to increases in Myosin-II contractility (43–45). When we treated WT, FMNL1 KO, and mDia1 KO T cells with nocodazole in low- and high-density collagen matrices, we found that migration was enhanced in all conditions, except for mDia1 KO T cells in high density collagen. This exception is surprising as migration was enhanced in mDia1 KO T cells treated with nocodazole in the low-density collagen, suggesting that mDia1 is sufficient, but not always necessary for enhancement of T cell motility upon microtubule destabilization. It is possible that, in addition to Myosin-II contractility, nocodazole-mediated GEF-H1 release from microtubules and subsequent RhoA activation promotes mDia1 activity, as RhoA has been proposed as an mDia1 activator (46, 47). However, the exact mechanism of this interaction and whether it is associated with mDia1’s actin polymerization or microtubule stabilization activity is not clear yet. Previous publications have shown that mDia1 is likely activated by RhoA binding, while FMNL1 is likely activated by Cdc42 or Rac1 binding (47, 56–59). This distinction may help explain why mDia1 KO T cell migration is not always enhanced by nocodazole, but FMNL1 KO T cell migration is. Recent publications have demonstrated that both the actin polymerization and microtubule stabilization activity of mDia1 are important for immune synapse formation in T cells (48, 60, 61). It has been shown that mDia1-mediated actin polymerization can be inhibited by the presence of microtubules (62). Thus, it’s possible that nocodazole-induced microtubule destabilization may be directly enhancing the actin polymerization kinetics of mDia1, explaining the varying interplay between nocodazole and migration enhancement in mDia1 sufficient and deficient T cells. However, it has also been shown that an mDia1/CLIP-170/EB1 complex can trigger faster actin polymerization at microtubule plus ends (63), suggesting that the actin polymerization-microtubule interaction is complex. Furthermore, nocodazole-induced microtubule destabilization has been shown to enhance nuclear deformability in T cells moving through a dense matrix (64). This phenomenon highlights an additional mechanism by which nocodazole can enhance T cell migration in 3D environments. This finding is also consistent with our data that FMNL1 KO T cells benefit from nocodazole treatment in terms of migration, as the increased nuclear deformability may be rescuing the defective nuclear deformation capacity in these FMNL1-deficent cells.

For T cells attempting to migrate through restrictive environments, movement of the nucleus through constrictions represents a rate-limiting step (65–67). Compared to the multi-lobed nucleus of neutrophils, the T cell nucleus is relatively rigid, necessitating a mechanism to deform it during migration through confined environments. Our previous study on FMNL1 in T cells found that when T cells encounter a constriction in *in vitro* fabricated microchannels, actin polymerization directly behind the nucleus increases dramatically when the nucleus engages the constriction (31). This increase was absent in FMNL1 KO T cells, leading us to investigate the nature of the interaction between formins and the nucleus. Here we found that FMNL1-deficiency impairs the T cell’s ability to deform its nucleus when moving through high density 3D environments. Furthermore, we found that FMNL1 physically associates with the nuclear compartment. Conversely, mDia1 is primarily cytoplasmic and is not required for nuclear deformation. While the subcellular fractionation shows that FMNL1 is associated with the nucleus, it is unknown if FMNL1 is interacting directly with the nuclear envelope via a post-translational modification or if there is an unidentified binding partner on the nuclear envelope. FHOD1, another member of the formin family expressed at low levels in T cells, has previously been shown to bind to nesprin proteins on the nuclear envelope in fibroblasts (68, 69). FMNL1 has also been shown to be regulated by n-myristoylation, a post-translational modification that facilitates and regulates subcellular structure targeting and membrane anchoring (70, 71). This raises two non-mutually exclusive possibilities for FMNL1 interactions with the nucleus. The first is that FMNL1 may have a protein binding partner on the nuclear envelope that couples the nucleus to the actin cytoskeleton to facilitate force transmission. The second is that n-myristoylation could be targeting FMNL1 to membranes to provide anchor points in general, with other T cell polarization mechanisms directing it to the posterior of the cell. It would be of interest in future studies to attempt to identify the nature of how FMNL1 is associated with the nuclear compartment.

High FMNL1 expression has been shown to be associated with more aggressive leukemia by promoting infiltration and has also been shown to be associated with metastasis in certain solid tumors (29, 72). Additionally, our previous work on FMNL1 demonstrated that FMNL1 deficient T cells have reduced capacity to induce T cell-mediated autoimmune disease (31). This makes FMNL1 an attractive target for disease intervention, particularly because FMNL1 tends to be restricted to hematopoietic lineage cells. Our data showing the distinct roles of FMNL1 and mDia1 also raises the possibility of mDia1 as a potential therapeutic target to exert further control over T cell migration. It will be of value in future work to determine how modulating specific formin expression or activity can fine-tune T cell infiltration and motility into peripheral tissues *in vivo* in various disease settings.

## Methods

### Mice

FMNL1 KO mice were generated as previously described (31). mDia1 KO mice are described in (32). LifeAct-GFP mice (73) were a kind gift of Dr. Roland Wedlich-Soldner (University of Munster). A list of key resources used in this article is provided in **Table 1**. All mice were bred and housed at the University of Colorado Anschutz Medical Campus Vivarium. Formin KO mice were paired with C57BL/6 mice that were age and sex matched. This study and mouse protocol were reviewed and approved by the Institutional Animal Care and Use Committees at the University of Colorado Anschutz Medical Campus, and all efforts were made to minimize mouse suffering.

**Table 1:** Key resources.

REAGENT or RESOURCE	SOURCE	IDENTIFIER
Antibodies		
Armenian Hamster anti-mouse CD3e (clone 2C11)	BioXCell	BE0001-1
Armenian Hamster anti-mouse CD28 (clone PV-1)	BioXCell	BE0015-5
Rabbit polyclonal anti-FMNL1	Invitrogen	PA5-52516
Goat polyclonal anti-FMNL1	Santa Cruz Biotech	
Mouse anti-mDia1 (clone 51)	BD Biosciences	610848
Rabbit anti-mDia1	ABClonal	A5772
Rabbit anti-lamin A/C (clone 4L8Q0)	Invitrogen	MA5-35284
Mouse anti-GAPDH (clone G-9)	Santa Cruz Biotech	Sc-365062
IRDye 680RD Donkey anti-Mouse IgG	Licor	926-68072
IRDye 800CW Donkey anti-Rabbit IgG	Licor	926-32213
Donkey anti-Goat IgG AlexaFluor Plus 555	Invitrogen	A32816
Donkey anti-Rabbit IgG AlexaFluor Plus 647	Invitrogen	A327795
Donkey anti-Mouse IgG AlexaFluor Plus 488	Invitrogen	A32766
Chemicals, peptides, and recombinant proteins		
Collagen I Rat Tail	Corning	354236
Recombinant Human IL-2	NCI	
InSolution Nocodazole	Millipore	487929
(S)-4’-nitro-Blebbistatin	Cayman Chemical	24171
CellTrace CFSE	Invitrogen	C34570
CellTrace Violet	Invitrogen	C34557
CellTrace Far Red	Invitrogen	C34564
Violet Proliferation Dye 450	BD Biosciences	562158
Hoechst 33342	Immunochemistry Technologies	639
Recombinant Mouse ICAM-1/CD54 Fc Chimera	R&D	796-IC-050
Recombinant Mouse IP-10 (CXCL10)	Peprotech	250-16
Critical commercial assays		
Subcellular Protein Fractionation Kit for Cultured Cells	Thermo Scientific	78840
Experimental models: Organisms/strains		
Mouse: WT C57BL/6J	The Jackson Laboratory	RRID : IMSR_JAX:000664
Mouse: FMNL1 KO C57BL/6J	Thompson et al. (31)	N/A
Mouse: mDia1 KO C57BL/6J	Sakata et al. (32)	N/A
Mouse: LifeAct-GFP	Riedl et al. (73)	RRID:IMSR_EM:12427
Software and algorithms		
ImageJ	Schindelin et al. (74)	https://imagej.net/software/fiji/#publication
Prism 10	GraphPad	https://www.graphpad.com/
MatLab	MathWorks	https://www.mathworks.com/products/matlab.html
Imaris 9	BitPlane	https://imaris.oxinst.com/
Other		
FluoSpheres Carboxylate-modified 0.2 µm dark red	Invitrogen	F8807
µ-Slide 8 Well high Glass Bottom	Ibidi	80807

### Primary T cell culture

Polyclonal T cells were harvested from C57BL/6 mouse superficial and mesenteric lymph nodes and activated using plate-bound anti-CD3 (2C11, BioXCell), soluble anti-CD28 (PV-1, BioXCell), and autologous splenocytes for two days. Cells were then removed from the stimulating antibodies and further cultured in RPMI (Corning) with 10% FBS, glutamine and antibiotics supplemented with 10 U/mL recombinant human IL-2 (NCI), refreshed every two days post-activation. On day four post-activation, dead cells were removed using a Histopaque-1110 (Sigma) gradient. Activated T cells were typically used for experiments on day 5 post-activation. With the stimulation conditions used, FMNL1 KO T cells and mDia1 KO T cells became activated and expanded similarly to WT T cells ( (31) and mDia1 KO data not shown).

### 2D T cell fixation and staining

T cells were seeded onto poly-lysine coated glass slides in phenol red-free RPMI supplemented with 20 mM HEPES, and 2% BSA and allowed to migrate freely at 37°C for 30-60 minutes. After this incubation, T cells were fixed in 4% PFA in PBS for 10 minutes. The cells were permeabilized with 1% Triton-X100 and blocked with 2% donkey serum. T cells were then stained with primary antibodies, washed several times, then stained with fluorophore-conjugated secondary antibodies. Slow Fade (ThermoFisher Scientific) was added prior to imaging.

### T cell dye labeling for microscopy

Ex vivo activated T cells were washed and re suspended in HBSS (Corning) at a concentration of 10×10^6^ cells/mL. Cells were labeled using CFSE, Cell Trace Violet (CTV), Cell Trace Far Red (CTFR) (all from Invitrogen), or Violet Proliferation Dye 450 (VPD) (BD Biosciences) at 37°C for 15 minutes and then washed multiple times. For nuclear deformation experiments, cells were stained with Hoechst 33342 (Immunochemistry Technologies) at 37°C for 15 minutes. Fluorescent cell dyes were rotated between genotypes in each experiment to control for potential toxicity.

### Collagen matrix preparation

Rat-Tail Type I collagen (Corning) was neutralized with 1 M NaOH and diluted with 10x PBS and sterile H_2_O. For nucleus deformation experiments, FluoSpheres 0.2 µm fluorescent microspheres were mixed into the collagen solution to visualize matrix density. Activated T cells were suspended at a concentration of 10x10^6/mL in phenol red-free RPMI supplemented with 20% FBS, 20 mM HEPES, and 2% BSA and mixed with neutralized collagen at a ratio of 3:1 (collagen mix:cell mix). CXCL10 (Peprotech) was then added to the mixture to a final concentration of 100 ng/mL. The presence of FBS (at a final 5% concentration) was included to improve the viability of the cells in the 3D collagen gels. For experiments utilizing cytoskeleton inhibitors, nocodazole was added to a final concentration of 2.5 μM and para-nitro-Blebbistatin at 3 μM or 10 μM. For vehicle controls, an equivalent volume of DMSO was added. The mixture was then mixed and pipetted into an 8-chamber slide (Ibidi) and the slide was then incubated at 37°C for 2-3 hours to allow the collagen to gel and the cells to adjust before imaging.

### Collagen fixation and staining

For immunofluorescence of T cells embedded in collagen, we adapted a published protocol for staining cells within intact collagen (75). After the collagen slide was incubated for 2-3 hours, a pre-warmed 37°C solution of 4% PFA and 5% sucrose in PBS was added to the surface of the hydrogel. The collagen was incubated at room temperature for 15 minutes after which the fixation solution was removed and the collagen blocks were gently transferred to tubes containing wash buffer (2% FBS, 0.01% sodium azide in PBS). The collagen gels were washed and stored in wash buffer at 4°C. The cells were then permeabilized with 0.1% Triton X100 (Sigma-Aldrich) for 10 minutes. After washing out the permeabilization buffer, the collagen gels were blocked in wash buffer supplemented with 3% donkey serum for 30 minutes. Primary antibodies were then added and incubated for 90 minutes with gentle mixing to ensure even penetration of the antibody mixture. After washing, secondary antibodies were added together with Hoechst 33342 and/or fluorescently-labelled Phalloidin (Invitrogen) and incubated in the dark for 1 hour, followed by several washes. The collagen gels were stored in fresh wash buffer at 4°C until ready to image.

### Cell lysis

For whole cell lysates, T cells were washed in PBS and then lysed with 1% Triton X100 buffer supplemented with Halt Protease and Phosphatase inhibitor cocktail (Thermo Scientific, Cat No. 78441). For subcellular fractionation experiments, the T cells were lysed in a series of subsequent steps according to the kit manufacturer instructions (Thermo Scientific, Cat No. 78840).

### Western blot

Cell lysates and subcellular fractions were run through homemade 7.5-18% polyacrylamide gradient gels and transferred to a nitrocellulose membrane. The membranes were briefly visualized using Ponceau S (Acros) to ensure proper transfer and blocked with SuperBlock PBS Blocking buffer (ThermoFisher). Membranes were stained and then imaged and analyzed using the Sapphire Biomolecular Imager (Azure Biosystems) and associated software. Whole cell lysates were normalized to GAPDH signal and subcellular fractions were normalized to the proportion of each fraction loaded.

### Confocal microscopy

For *in vitro* confocal imaging experiments, we used a Zeiss LSM 800 Confocal Laser Scanning Microscope. For live cell imaging, samples were kept at 37°C using a stage heater. Bulk motility experiments were captured using a 10X objective and high-resolution single-cell analysis experiments were captured using a 40X water objective. Images were processed using ImageJ (NIH) and motility parameters were analyzed using Imaris software (Bitplane) as described below. For fixed cell imaging on 2D surfaces, slides prepared as described above were imaged using a 63X oil objective. For imaging fixed cells in collagen, the samples prepared as described above were transferred to 8-well glass bottom chamber slides and imaged using a 40X water objective. Time lapse microscopy was performed by repeated XY images with a Z-step of 3 µm every 30s for 25-30 minutes.

### Image analysis of fixed cells *in vitro*

Fixed cell images were analyzed using ImageJ (NIH). Images were pre-processed by generating a sum z-projection. For each individual polarized T cell, front and back regions were manually defined relative to the nucleus based on morphological features. These regions were then used to define masks to calculate the total intensity of FMNL1 and mDia1 in each region. The front and back regions of the cell were determined in a blinded way in relation to the distribution of FMNL1 and mDia1. For 3D experiments, only polarized cells that were oriented primarily in the XY plane were analyzed.

### Image analysis of live cells *in vitro*

Analysis of T cell motility parameters was done using Imaris (Bitplane) to automatically track cells, followed by manual correction of tracking errors that resulted in broken tracks. For the analysis of T cells in 3D collagen gels, only cells that were fully embedded in the collagen gels were imaged. This was ensured by starting the Z stacks at least 40 μm above the glass coverslip. Immobile cells with no cytoplasmic motion were assumed dead and removed from the analysis, a similar percentage of cells for each genotype was excluded by this criterion. The parameters quantified were mean track speed, arrest coefficient (% of timepoints a cell’s instantaneous speed was less than 2 µm/min), and mean squared displacement. Data were filtered to include only cells tracked for at least 4 min. Mean track speed was also filtered to include cells with a minimum displacement of 15 µm. For experiments using para-nitro-Blebbistatin, tracks were filtered only by a minimum duration of 4 min. For experiments quantifying nuclear deformation, imaging time points where the nuclear surface was partially out of field were manually removed.

### Time-dependent sequential Bayesian inference of heterogenous 3D random walk

The velocity vector $u_{t}=\left(u_{x},u_{y},u_{z}\right)$ representing the 3D cell motion at a time t from the origin was numerically calculated with a 2^nd^ order centered finite difference scheme for all trajectories. Adapting the methodology of Metzner et al. (38), the stochastic change in the velocity of a cell was modeled as a random walk (first order autoregressive process) given by $u_{t}=\;q_{t}\;u_{t-1}+\;a_{t}n_{t}$ . Here, q_t_ is a number in the range [-1,1] representing the instantaneous persistence of the 3D random walk and a_t_ is a number in the range [0,∞] representing the instantaneous activity that sets the amplitude of the random walk. The random nature of the process is reflected by n_t_, representing normally distributed noise with unit variance and zero temporal memory. The parameters (q_t_, a_t_) are time-dependent for the random walk to be heterogeneous. The parameter q_t_ is a measure of the directional correlation between successive time steps with (q_t_=1, a_t_=0) corresponding to motion with constant speed along a perfectly straight trajectory, and q_t_=0 corresponding to purely Brownian. Higher values of a_t_ correlate with increases in metrics such as cell speed, total displacement, and track length. These parameters can be inferred from tracked trajectory data using Bayesian sequential inference (38). The analysis was performed in MATLAB to estimate the heterogenous migration parameters. Since the random walk model is Markovian with finite time interval, i.e. $u_{t}$ . conditioned on $u_{t-1}$ only, inferring one set of parameters $\left(q_{t},a_{t}\right)$ requires two consecutive velocity measurements, each of which in-turn require three consecutive position measurements. Any gap in the trajectory invalidates neighboring velocity estimates and for reliable estimation of random walk parameters, only cells with at least 15 valid velocity measurements were considered. The MATLAB scripts used for this analysis can be found at: https://github.com/adithank/ClusterRandomWalk-SiglerEtAl

### Semi-unsupervised identification of heterogenous sub-populations in migrating T cells

Each migrating cell (tracked for at least 240 s) is characterized by the average random walk parameters (q, a) of persistence and activity obtained from sequential Bayesian inference (38). These parameters represent the average motility state of the migrating T cell in relation to a persistent random walk model. The density-based clustering DBSCAN algorithm (76) was used to detect outliers. After outlier removal, k-means clustering was employed to detect clusters in the (q, a) parameter space of all cells. Using silhouette width (77) as a reference guide and bootstrapping as a measure of stability, k=4 was selected for k-means clustering. The mean silhouette scores for k=2,3,4 were comparable, indicating that similar clustering accuracies would be obtained by allocations containing 2, 3 or 4 clusters. However, inspecting the probability distributions of (q, a) (**Supplementary Figures 2B–G**) indicated that k=4 clusters reflected these distributions optimally across different conditions by identifying a (↑a↑q) sub-population, a (↓a↑q) sub-population, and two (↓a↓q) sub-populations, one of which consisted of essentially non-motile cells. Therefore, each cell was assigned to one of the 4 clusters that minimized the Euclidean distance to the cluster centroid, cells belonging to either one of the two (↓a↓q) sub-populations were merged into a single (↓a↓q) sub-population, and the proportions of the three sub-populations: (↑a↑q), (↓a↑q), and (↓a↓q), were reported. Bootstrap analysis was performed to assess the stability of the clustering solution (78), providing mean and standard deviation of cluster centroids. The bootstrap analysis resamples the data points (i.e. (q, a) average of a cell) with replacement and performs the clustering procedure described above resulting in cluster proportions. This bootstrap procedure was repeated 100 times and the mean of cumulative sum of the cluster proportions are reported. This clustering with the bootstrap procedure was adapted independently for the para-nitro-Blebbistatin and nocodazole inhibitor treatment experiments, for which a k=3 clusters provided the best fit.

### 2D contour plot generation

The 3D persistent random walk parameter space is discretized in the domain a∈0.5µ^2^/min and q∈[0,1] encompassing the results of sequential Bayesian inference of heterogenous random walk. Each parameter (a_t_, q_t_) represents the random walk behavior describing two successive velocity vectors from a cell trajectory. All such pairs of parameters (a_t_, q_t_) within an experimental condition and collagen concentration is used as input to *histcounts2* function in MATLAB to produce bivariate histograms. The resulting 2D histogram is plotted as 2D contour plots with three levels that contains 50%, 80% and 95% of the joint probability mass. 1D histogram projections of the corresponding variables are plotted to the top and the right.

### Transwell assay

Transwell assay plates were prepared by adding 600 μL of RPMI supplemented with 2% (wt/vol) BSA and 10mM HEPES buffer, with 100ng/mL CXCL10. Then 5x10^5^ WT, FMNL1 KO, or mDia1 KO T cells were added to the top chambers and allowed to migrate for 1 h at 37°C into the lower well. As a standard to calculate the percentage of migrated cells, 5x10^5^ cells (20% of input cells added to transwells) were placed directly into bottom wells with no transwell. Each condition was set up in duplicate. Migrated T cells were collected by gently pipetting from the bottom wells and 25 μL of CountBright Absolute Counting Beads (ThermoFisher Scientific) were added to each sample to enable counting of migrated cells. Samples were then quantified on a Cytek Aurora flow cytometer for a set amount of time. The number of cells counted was normalized to the number of beads counted to adjust for any variations in flow rate during the run. For Myosin-II inhibition experiments, T cells were incubated for 30 min at 37°C with 100 μM Blebbistatin (Millipore Sigma) or an equivalent amount of DMSO vehicle prior to the transwell assay. During the assay, this concentration was maintained in both transwell chambers, as well as the 20% standard wells used to calculate the percentage of migrated cells.

### *In vivo* motility by two-photon microscopy

Ex vivo activated WT, FMNL1 KO, or mDia1 KO T cells were dye labelled as described above and a 1:1 mix of WT and KO cells was transferred into WT recipient mice by tail vein injection. Between 18-24 hours after transfer, the mice were euthanized and their LNs were harvested for imaging. Explanted LNs were immobilized with the efferent lymphatics adhered to the coverslip using Vetbond (3M). The LNs were placed in a flow chamber (Warner instruments PH-1) and perfused with RPMI medium without phenol red (Gibco) saturated with 95% O_2_ and 5% CO_2_ and kept at 35–37°C. Two-photon imaging was performed using a Leica SP8 DIVE upright two-photon microscope with a SpectraPhysics InsightX3 dual line (tunable 680-1300nm and 1045nm) IR laser with pre-chirp compensation, 4 tunable spectral non-descanned detectors, galvanometer confocal scanner and high-speed resonant confocal scanner. Time-lapse imaging was done by repeated imaging of XY planes of 512x512 pixels at 1.16μm/pixel and Z-steps of 3μm with XYZ stacks acquired every ~30 sec for ~30 min. The acquired images were analyzed using Imaris software (Bitplane). To eliminate any potential bleed-over between channels the images were linearly unmixed using the Channel Arithmetic function in Imaris to subtract any non-specific fluorescence bleeding from one channel to the other as previously described (79). Cells in lymph nodes that could be tracked for ≥5 min were included in the analysis and average speed, turning angle distribution, arrest coefficient (time spent migrating at <2 µm/min), and mean square displacement (MSD) were calculated.

## Data Availability

The raw data supporting the conclusions of this article will be made available by the authors, without undue reservation.

## Supplementary material

Supplementary Figure 1 Formin family mRNA expression in naïve and activated T cells. CD8 T cells were harvested and purified from either WT or OT-I donor mice. Formin expression was analyzed by quantitative PCR for naive WT CD8 T cells **(A)** and activated OT-I T cells **(B)**. Expression was normalized to the formin with the lowest expression, in this case FHOD3. Data represents the mean +/- SEM for three technical replicates within one independent experiment.

Supplementary Figure 2 Persistent random walk clustering analysis and displacement analysis by experiment of T cells in 3D collagen matrices. WT, FKO, and mDKO T cells trajectories from **Figure 4** were fit to a persistent random walk model and activity and persistence values were calculated. **(A)** Cluster map with cell averaged 3D persistent random walk parameters (a,q). K-means clustering with k=4 was performed (left) and non-motile cells were pooled with the ↓a↓q cluster for further analysis (right). **(B–G)** 2D contour maps representing probability distributions of cell averaged a,q values. Maps are overlaid to allow for visual comparison between WT, FKO, and mDKO cells within low- or high-density collagen. **(H-I)** Analysis of mean squared displacement averages by experiment for WT, FKO, and mDKO T cells in 0.5 mg/mL **(H)** or 2.0 mg/mL **(I)** collagen matrices comparing the different genotypes from **Figure 4**. Each point represents one biological replicate. Lines connect replicates obtained from the same experiment. Significance was determined by ratio paired t-test. ns, not significant.

Supplementary Figure 3 Persistent random walk clustering analysis and displacement analysis by experiment of vehicle and para-nitro-Blebbistatin treated T cells. WT, FKO, and mDKO T cell trajectories from **Figures 5D–F** were fit to a persistent random walk model and activity and persistence values were calculated. **(A)** Scatter plots showing the overlay of DMSO (grey) or 10 μM para-nitro-blebbistatin (red) treated WT, FKO, and mDKO T cells and corresponding stacked bar plots representing the proportion of DMSO and para-nitro-blebbistatin treated T cells clustering to three modes of motility described in **Figure 4** and **Supplementary Figure 2**. **(B)** Analysis of mean squared displacement averages by experiment for WT, FKO, and mDKO in 1.0 mg/mL collagen matrices treated with either DMSO or 10 μM para-nitro-blebbistatin comparing the different genotypes from data in **Figures 5D–F**. Each point represents one biological replicate. Lines connect replicates obtained from the same experiment. Significance in **(A)** was determined by a two-population proportion Z test. Significance in **(B)** was determined by ratio paired t-test. ns, not significant, ** = p<0.01, *** = p<0.001.

Supplementary Figure 4 Nocodazole dose titration on WT T cells in 3D collagen. WT T cells were collected from donor mice and *ex vivo* activated. Cells were stained with CFSE and embedded in 2.0 mg/mL collagen matrices with increasing concentrations of Nocodazole (2.5, 10, 40 μM) or an equivalent amount of DMSO as a vehicle control. **(A–C)** Quantification of mean track speed **(A)**, arrest coefficient **(B)**, and mean square displacement of cells tracked continuously for 10 minutes **(C)**. Data represents the mean +/- SEM for >=79 cells per condition from one experiment. Significance was determined by Brown-Forsythe and Welch ANOVA tests with Games-Howell’s multiple comparisons test. ns, not significant, * = p<0.05, *** = p<0.001, **** = p<0.0001.

Supplementary Figure 5 Persistent random walk analysis and displacement analysis by experiment of vehicle and nocodazole treated T cells. WT, FKO, and mDKO T cell trajectories in 2.0 mg/mL collagen matrices from **Figure 6** were fit to a persistent random walk model and activity and persistence values were calculated. **(A)** Scatter plots showing the overlay of DMSO (red) or 2.5 μM nocodazole (blue) treated WT, FKO, and mDKO T cells and corresponding stacked bar plots representing the proportion of DMSO and nocodazole treated T cells clustering to the three modes of motility described in **Figure 4** and **Supplementary Figure 2**. **(B)** Analysis of mean squared displacement averages by experiment for WT, FKO, and mDKO T cells in 2.0 mg/mL collagen matrices treated with either DMSO or 2.5 μM nocodazole comparing the treatment conditions within each genotype from **Figure 6**. Each point represents one biological replicate. Lines connect replicates obtained from the same experiment. Significance in **(A)** was determined by a two-population proportion Z test. Significance in **(B)** was determined by ratio paired t-test. ns, not significant, *** = p<0.001.

Supplementary Figure 6 Control T cells have a high dynamic range of nuclear ellipticity. Control T cells were collected from donor WT or LifeAct-GFP expressing donor mice and *ex vivo* activated. The cells were stained with Hoechst nuclear dye and embedded in 2.0 mg/mL collagen matrices seeded with fluorescent microspheres. T cell cortex and cytoplasm was visualized using either LifeAct-GFP or an additional CFSE dye labeling step. These data represent a subset of control cells from **Figure 7**. **(A)** Maximum and minimum prolate ellipticity values of control cells migrating in 2.0 mg/mL collagen matrices. **(B)** Prolate ellipticity values over time for the control cell represented in **Figure 7A**. Pre-, Mid-, and Post-constriction points were determined by quantitative and qualitative features. Pre- and post-constriction points were determined by identifying local minima values of prolate ellipticity. The mid-constriction point was determined by the local maximum value of prolate ellipticity in combination with the image frame where the cell is maximally engaged with the constriction visualized by the fluorescent microspheres. Data in **(A)** represent the mean +/- SEM of 37 cells pooled from three independent experiments, including a subset of control cells used in **Figure 7**. Significance was determined by unpaired t-test.

Supplementary Figure 7 Graphical model of formin-mediated T cell migration in 3D environments Illustration of a T cell encountering a restriction point in a 3D collagen environment. We propose a model of formin-mediated T cell migration in which FMNL1 and mDia1 have distinct roles. Our data suggests that FMNL1 is localized to the rear of the T cell and promotes deformation of the rigid nucleus independently of Myosin II via physical association with the nuclear compartment. Conversely, mDia1 is distributed more evenly throughout the cell and is almost entirely cytoplasmic, promoting general T cell motility in conjunction with Myosin II activity. Created with BioRender.com

Supplementary Movie 1 WT and FMNL1 KO T cells were collected from donor mice and activated *ex vivo*. The T cells were then differentially dye-labeled with CFSE or VPD and co-transferred at a 1:1 ratio into WT recipient mice. 18-24 hours later, lymph nodes were harvested from the recipient mice and analyzed by two-photon microscopy. FMNL1 KO (green) and WT (red) T cells were tracked for ~30 minutes. The tracks show the path of T cells over the prior 10 minutes. Time stamp is in minutes:seconds.

Supplementary Movie 2 WT and mDia1 KO T cells were collected from donor mice and activated *ex vivo*. The T cells were then differentially dye-labeled with CFSE or VPD and co-transferred at a 1:1 ratio into WT recipient mice. 18-24 hours later, lymph nodes were harvested from the recipient mice and analyzed by two-photon microscopy. mDia1 KO (green) and WT (red) T cells were tracked for ~30 minutes. The tracks show the path of T cells over the prior 10 minutes. Time stamp is in minutes:seconds.

Supplementary Movie 3 WT, FMNL1 KO, and mDia1 KO T cells were collected from donor mice and activated *ex vivo*. The cells were differentially dye-labeled with CFSE, CTV, or CTFR, and seeded onto an ICAM-1 coated glass slide. After 1 hour, the cells were analyzed by time-lapse confocal microscopy. Individual WT (green), FMNL1 KO (cyan), and mDia1 KO (red) T cells were tracked over 15 minutes. Track lines show the path of the T cells taken over the prior 10 minutes. Scale bar = 50 μm.

Supplementary Movie 4 WT, FMNL1 KO, and mDia1 KO T cells were collected from donor mice and activated *ex vivo*. The cells were differentially dye-labeled with CFSE, CTV, or CTFR and embedded in a 0.5 mg/mL collagen matrix with 100 ng/mL CXCL10. After incubating at 37°C for 2 hours, the cells were analyzed by time-lapse confocal microscopy. Individual WT (green), FMNL1 KO (cyan), and mDia1 KO (red) T cells were tracked over 30 minutes. Track lines show the path of the T cells taken over the prior 10 minutes. Scale bar = 50 μm.

Supplementary Movie 5 WT, FMNL1 KO, and mDia1 KO T cells were collected from donor mice and activated *ex vivo*. The cells were differentially dye-labeled with CFSE, CTV, or CTFR and embedded in a 2.0 mg/mL collagen matrix with 100 ng/mL CXCL10. After incubating at 37°C for 2 hours, the cells were analyzed by time-lapse confocal microscopy. Individual WT (green), FMNL1 KO (cyan), and mDia1 KO (red) T cells were tracked over 30 minutes. Track lines show the path of the T cells taken over the prior 10 minutes. Scale bar = 50 μm.

Supplementary Movie 6 WT, FMNL1 KO, and mDia1 KO T cells were collected from donor mice and activated *ex vivo*. The cells were differentially dye-labeled with CFSE, CTV, or CTFR and embedded in a 1.0 mg/mL collagen matrix with 100 ng/mL CXCL10 and a DMSO vehicle control. After incubating at 37°C for 2 hours, the cells were analyzed by time-lapse confocal microscopy. Individual WT (red), FMNL1 KO (green), and mDia1 KO (cyan) T cells were tracked over 25 minutes. Track lines show the path of the T cells taken over the prior 10 minutes. Scale bar = 50 μm.

Supplementary Movie 7 WT, FMNL1 KO, and mDia1 KO T cells were collected from donor mice and activated *ex vivo*. The cells were differentially dye-labeled with CFSE, CTV, or CTFR and embedded in a 1.0 mg/mL collagen matrix with 100 ng/mL CXCL10 and 10 μM para-nitro-Blebbistatin. After incubating at 37°C for 2 hours, the cells were analyzed by time-lapse confocal microscopy. Individual WT (red), FMNL1 KO (green), and mDia1 KO (cyan) T cells were tracked over 25 minutes. Track lines show the path of the T cells taken over the prior 10 minutes. Scale bar = 50 μm.

Supplementary Movie 8 WT, FMNL1 KO, and mDia1 KO T cells were collected from donor mice and activated *ex vivo*. The cells were differentially dye-labeled with CFSE, CTV, or CTFR and embedded in a 0.5 mg/mL collagen matrix with 100 ng/mL CXCL10 and a DMSO vehicle control. After incubating at 37°C for 2 hours, the cells were analyzed by time-lapse confocal microscopy. Individual WT (cyan), FMNL1 KO (red), and mDia1 KO (green) T cells were tracked over 25 minutes. Track lines show the path of the T cells taken over the prior 10 minutes. Scale bar = 50 μm.

Supplementary Movie 9 WT, FMNL1 KO, and mDia1 KO T cells were collected from donor mice and activated *ex vivo*. The cells were differentially dye-labeled with CFSE, CTV, or CTFR and embedded in a 0.5 mg/mL collagen matrix with 100 ng/mL CXCL10 and 2.5 μM nocodazole. After incubating at 37°C for 2 hours, the cells were analyzed by time-lapse confocal microscopy. Individual WT (red), FMNL1 KO (green), and mDia1 KO (cyan) T cells were tracked over 25 minutes. Track lines show the path of the T cells taken over the prior 10 minutes. Scale bar = 50 μm.

Supplementary Movie 10 WT, FMNL1 KO, and mDia1 KO T cells were collected from donor mice and activated *ex vivo*. The cells were differentially dye-labeled with CFSE, CTV, or CTFR and embedded in a 2.0 mg/mL collagen matrix with 100 ng/mL CXCL10 and a DMSO vehicle control. After incubating at 37°C for 2 hours, the cells were analyzed by time-lapse confocal microscopy. Individual WT (red), FMNL1 KO (green), and mDia1 KO (cyan) T cells were tracked over 25 minutes. Track lines show the path of the T cells taken over the prior 10 minutes. Scale bar = 50 μm.

Supplementary Movie 11 WT, FMNL1 KO, and mDia1 KO T cells were collected from donor mice and activated *ex vivo*. The cells were differentially dye-labeled with CFSE, CTV, or CTFR and embedded in a 2.0 mg/mL collagen matrix with 100 ng/mL CXCL10 and 2.5 μM nocodazole. After incubating at 37°C for 2 hours, the cells were analyzed by time-lapse confocal microscopy. Individual WT (red), FMNL1 KO (green), and mDia1 KO (cyan) T cells were tracked over 25 minutes. Track lines show the path of the T cells taken over the prior 10 minutes. Scale bar = 50 μm.
