# Supplementary figures and images for: FMNL1 and mDia1 promote efficient T cell migration through complex environments via distinct mechanisms
Source: Front Immunol. 2024 Oct 4;15:1467415. doi: 10.3389/fimmu.2024.1467415 (PMC11486666; doi:10.3389/fimmu.2024.1467415)

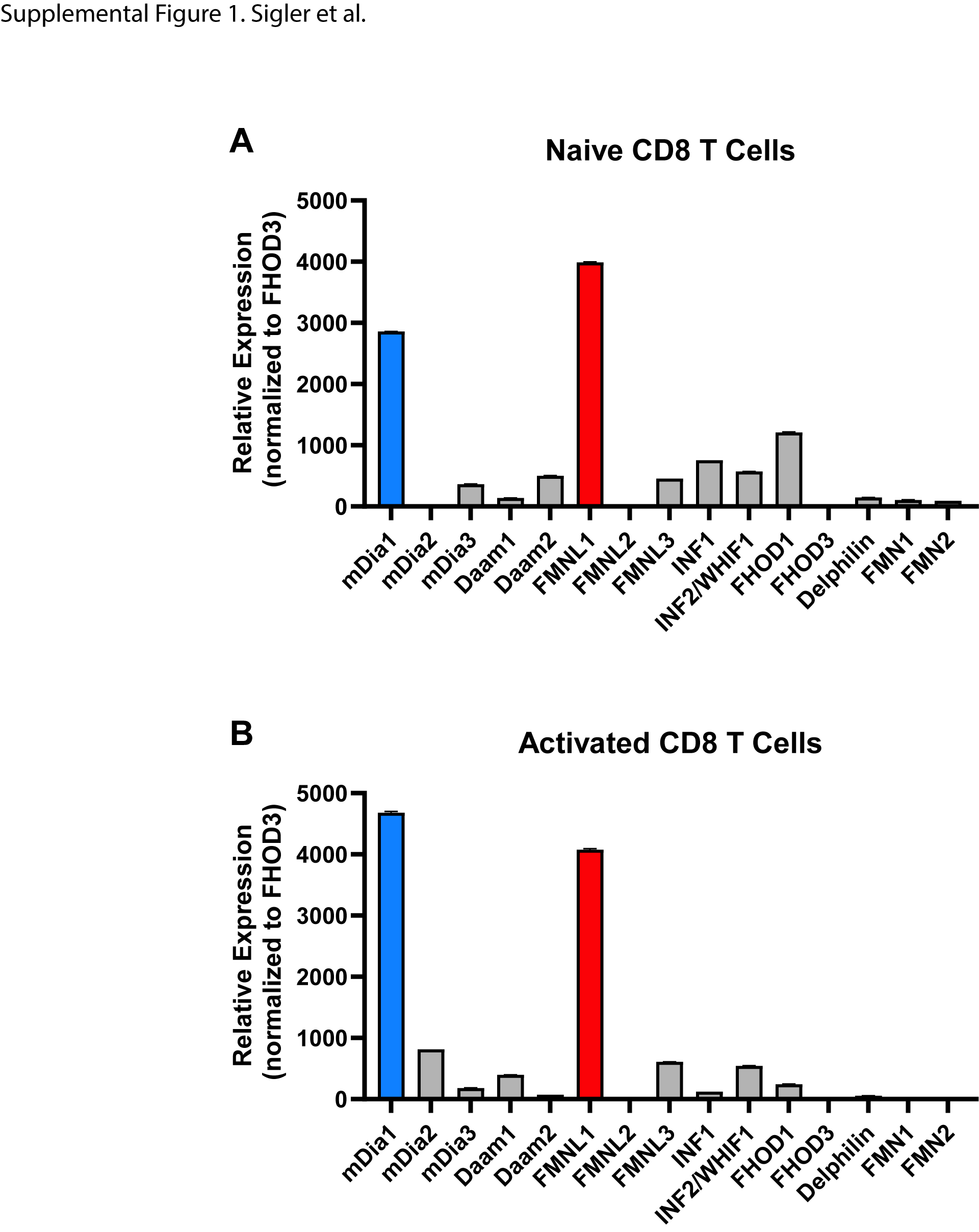

Supplement: Supplementary Figure 1 — Formin family mRNA expression in naïve and activated T cells. CD8 T cells were harvested and purified from either WT or OT-I donor mice. Formin expression was analyzed by quantitative PCR for naive WT CD8 T cells (A) and activated OT-I T cells (B). Expression was normalized to the formin with the lowest expression, in this case FHOD3. Data represents the mean +/- SEM for three technical replicates within one independent experiment. [file Image1.tif]

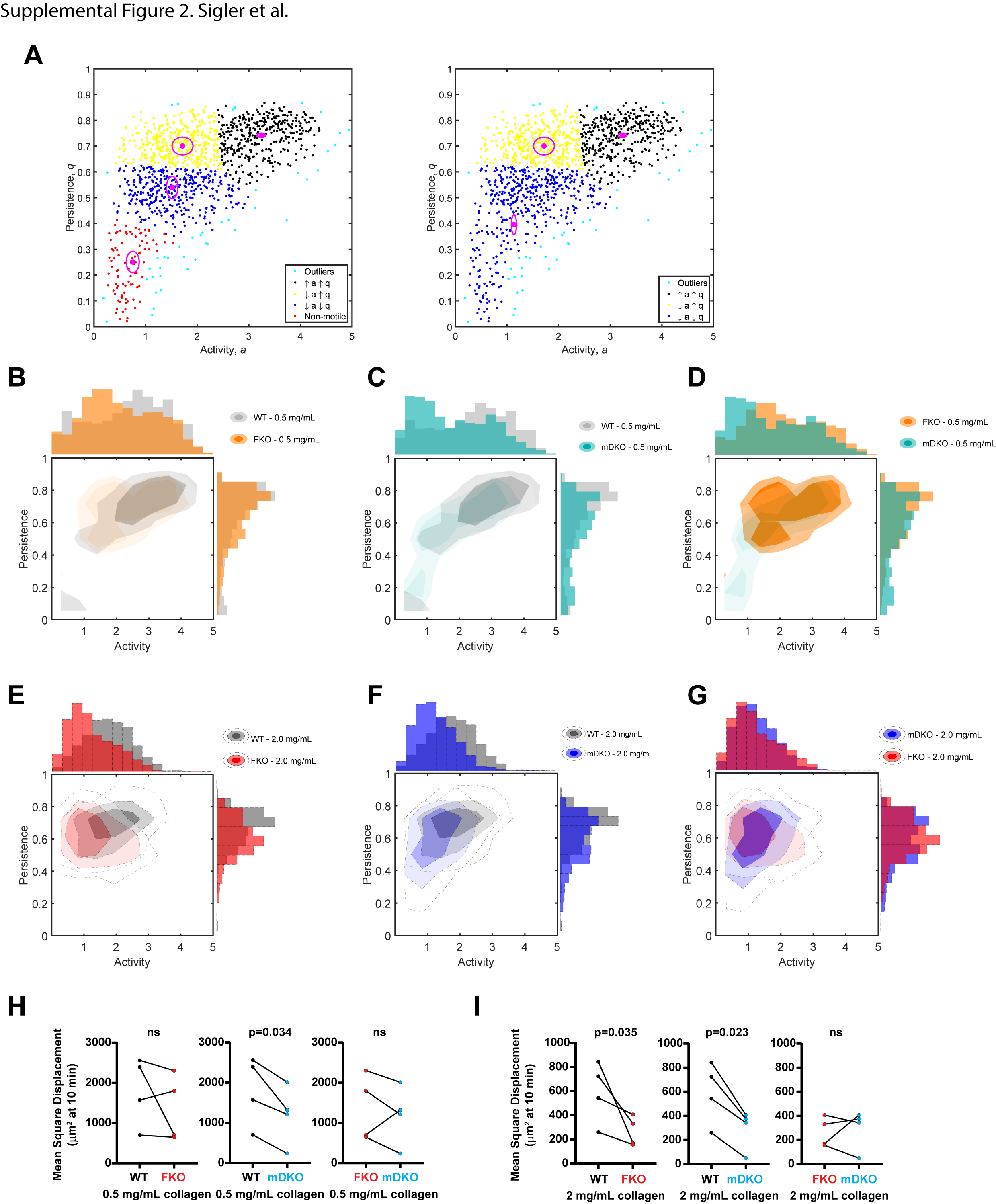

Supplement: Supplementary Figure 2 — Persistent random walk clustering analysis and displacement analysis by experiment of T cells in 3D collagen matrices. WT, FKO, and mDKO T cells trajectories from Figure 4 were fit to a persistent random walk model and activity and persistence values were calculated. (A) Cluster map with cell averaged 3D persistent random walk parameters (a,q). K-means clustering with k=4 was performed (left) and non-motile cells were pooled with the ↓a↓q cluster for further analysis (right). (B–G) 2D contour maps representing probability distributions of cell averaged a,q values. Maps are overlaid to allow for visual comparison between WT, FKO, and mDKO cells within low- or high-density collagen. (H-I) Analysis of mean squared displacement averages by experiment for WT, FKO, and mDKO T cells in 0.5 mg/mL (H) or 2.0 mg/mL (I) collagen matrices comparing the different genotypes from Figure 4 . Each point represents one biological replicate. Lines connect replicates obtained from the same experiment. Significance was determined by ratio paired t-test. ns, not significant. [file Image2.tif]

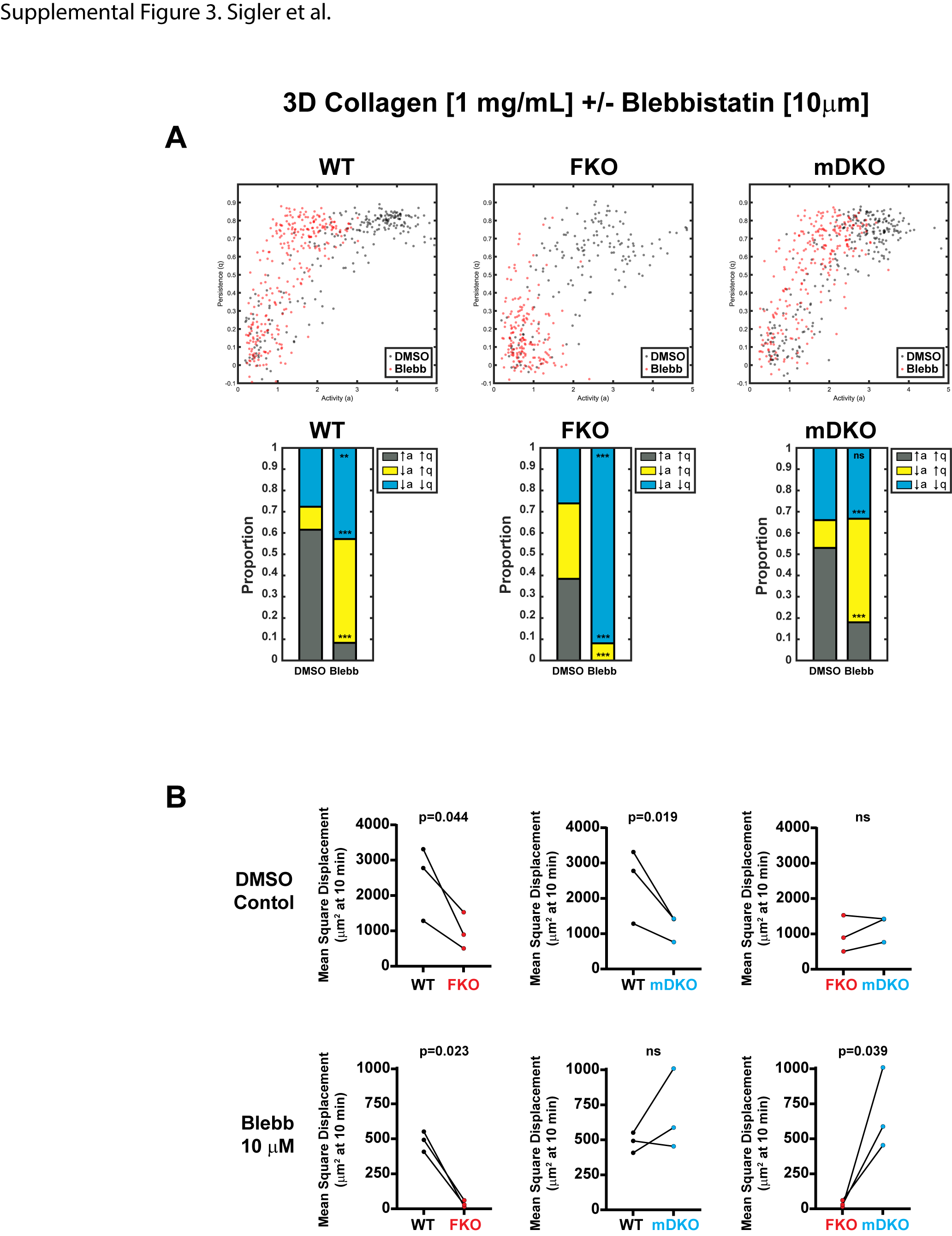

Supplement: Supplementary Figure 3 — Persistent random walk clustering analysis and displacement analysis by experiment of vehicle and para-nitro-Blebbistatin treated T cells. WT, FKO, and mDKO T cell trajectories from Figures 5D–F were fit to a persistent random walk model and activity and persistence values were calculated. (A) Scatter plots showing the overlay of DMSO (grey) or 10 μM para-nitro-blebbistatin (red) treated WT, FKO, and mDKO T cells and corresponding stacked bar plots representing the proportion of DMSO and para-nitro-blebbistatin treated T cells clustering to three modes of motility described in Figure 4 and Supplementary Figure 2 . (B) Analysis of mean squared displacement averages by experiment for WT, FKO, and mDKO in 1.0 mg/mL collagen matrices treated with either DMSO or 10 μM para-nitro-blebbistatin comparing the different genotypes from data in Figures 5D–F . Each point represents one biological replicate. Lines connect replicates obtained from the same experiment. Significance in (A) was determined by a two-population proportion Z test. Significance in (B) was determined by ratio paired t-test. ns, not significant, ** = p<0.01, *** = p<0.001. [file Image3.tif]

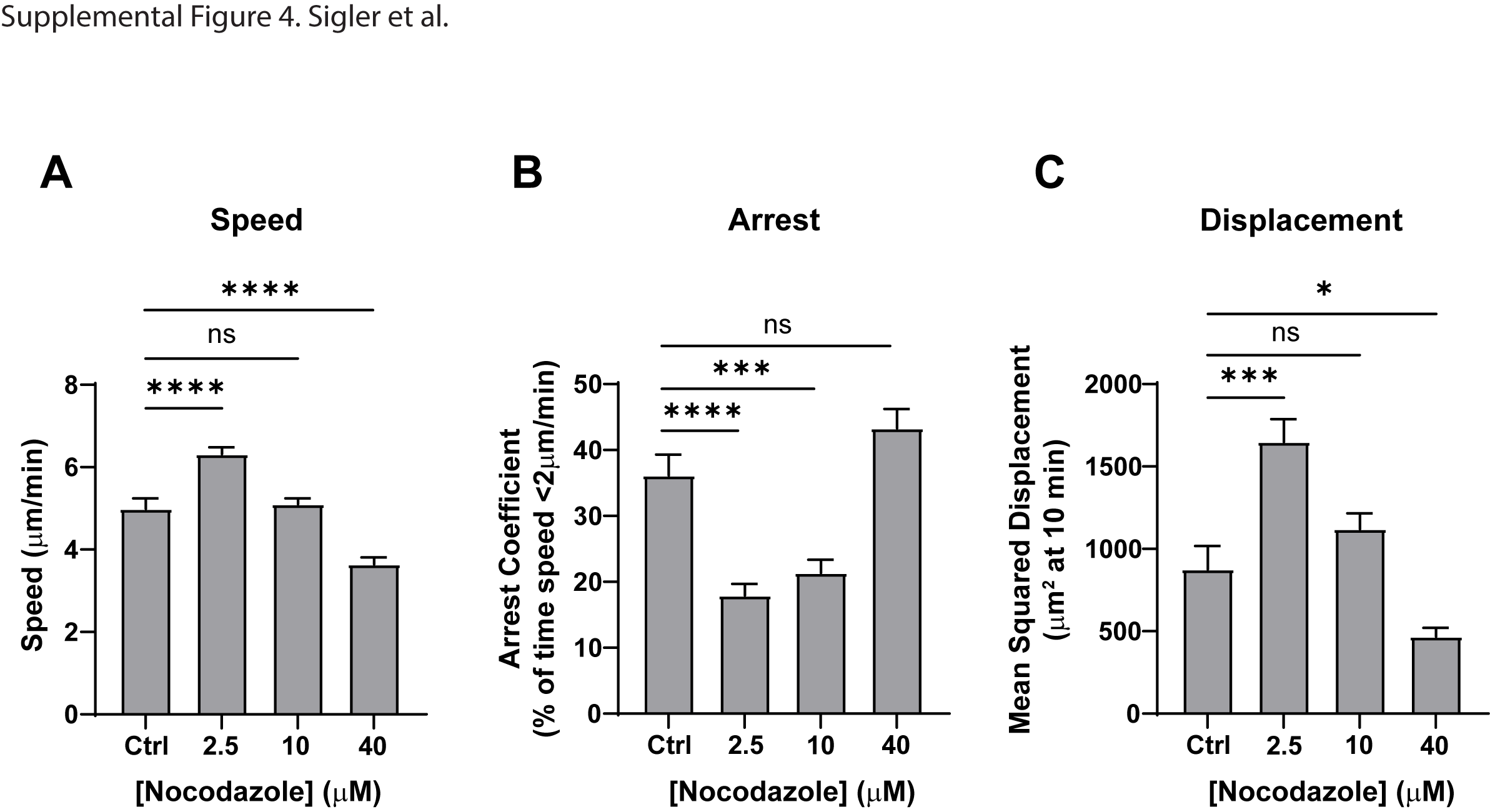

Supplement: Supplementary Figure 4 — Nocodazole dose titration on WT T cells in 3D collagen. WT T cells were collected from donor mice and ex vivo activated. Cells were stained with CFSE and embedded in 2.0 mg/mL collagen matrices with increasing concentrations of Nocodazole (2.5, 10, 40 μM) or an equivalent amount of DMSO as a vehicle control. (A–C) Quantification of mean track speed (A), arrest coefficient (B), and mean square displacement of cells tracked continuously for 10 minutes (C). Data represents the mean +/- SEM for >=79 cells per condition from one experiment. Significance was determined by Brown-Forsythe and Welch ANOVA tests with Games-Howell’s multiple comparisons test. ns, not significant, * = p<0.05, *** = p<0.001, **** = p<0.0001. [file Image4.tif]

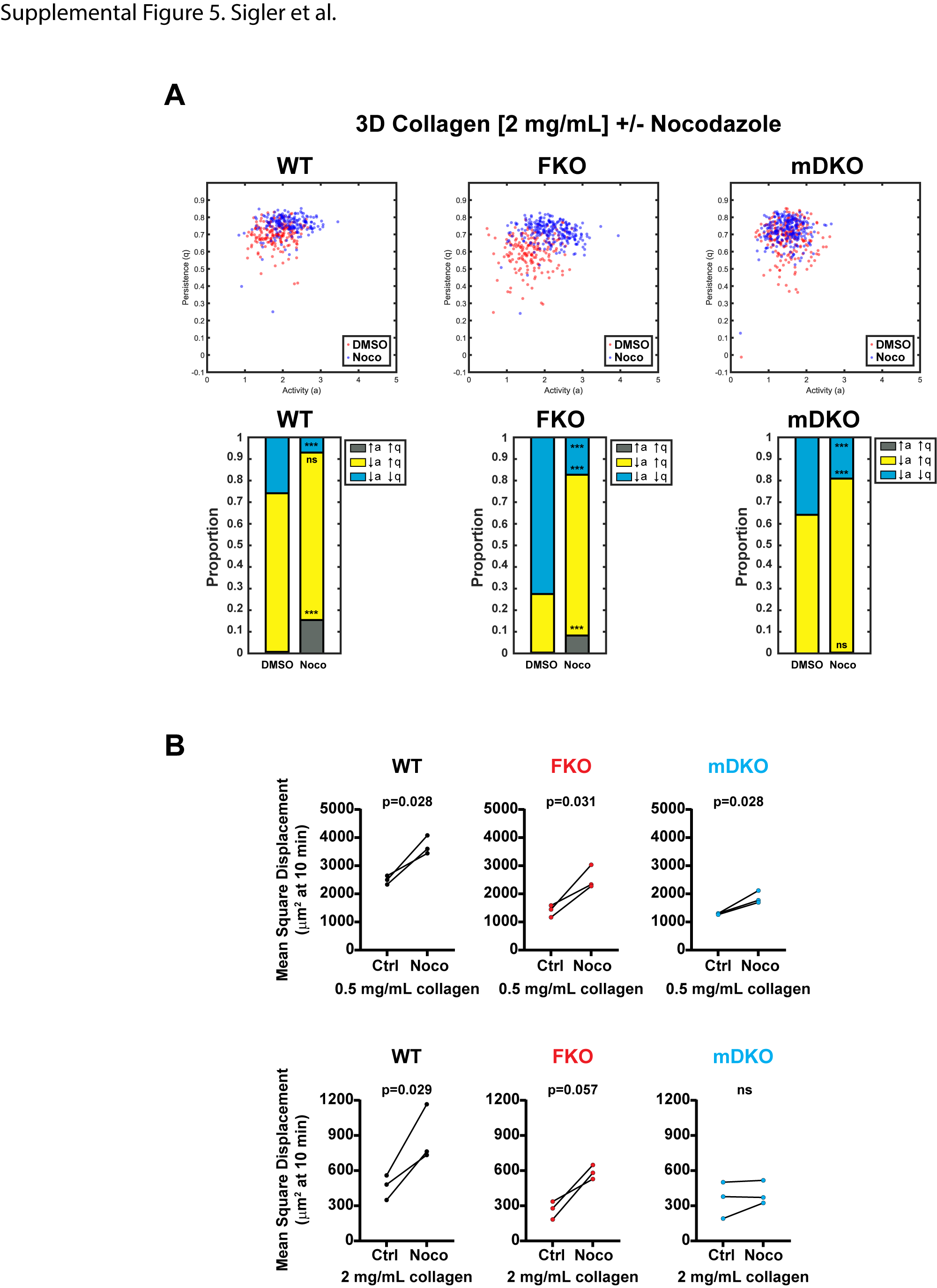

Supplement: Supplementary Figure 5 — Persistent random walk analysis and displacement analysis by experiment of vehicle and nocodazole treated T cells. WT, FKO, and mDKO T cell trajectories in 2.0 mg/mL collagen matrices from Figure 6 were fit to a persistent random walk model and activity and persistence values were calculated. (A) Scatter plots showing the overlay of DMSO (red) or 2.5 μM nocodazole (blue) treated WT, FKO, and mDKO T cells and corresponding stacked bar plots representing the proportion of DMSO and nocodazole treated T cells clustering to the three modes of motility described in Figure 4 and Supplementary Figure 2 . (B) Analysis of mean squared displacement averages by experiment for WT, FKO, and mDKO T cells in 2.0 mg/mL collagen matrices treated with either DMSO or 2.5 μM nocodazole comparing the treatment conditions within each genotype from Figure 6 . Each point represents one biological replicate. Lines connect replicates obtained from the same experiment. Significance in (A) was determined by a two-population proportion Z test. Significance in (B) was determined by ratio paired t-test. ns, not significant, *** = p<0.001. [file Image5.tif]

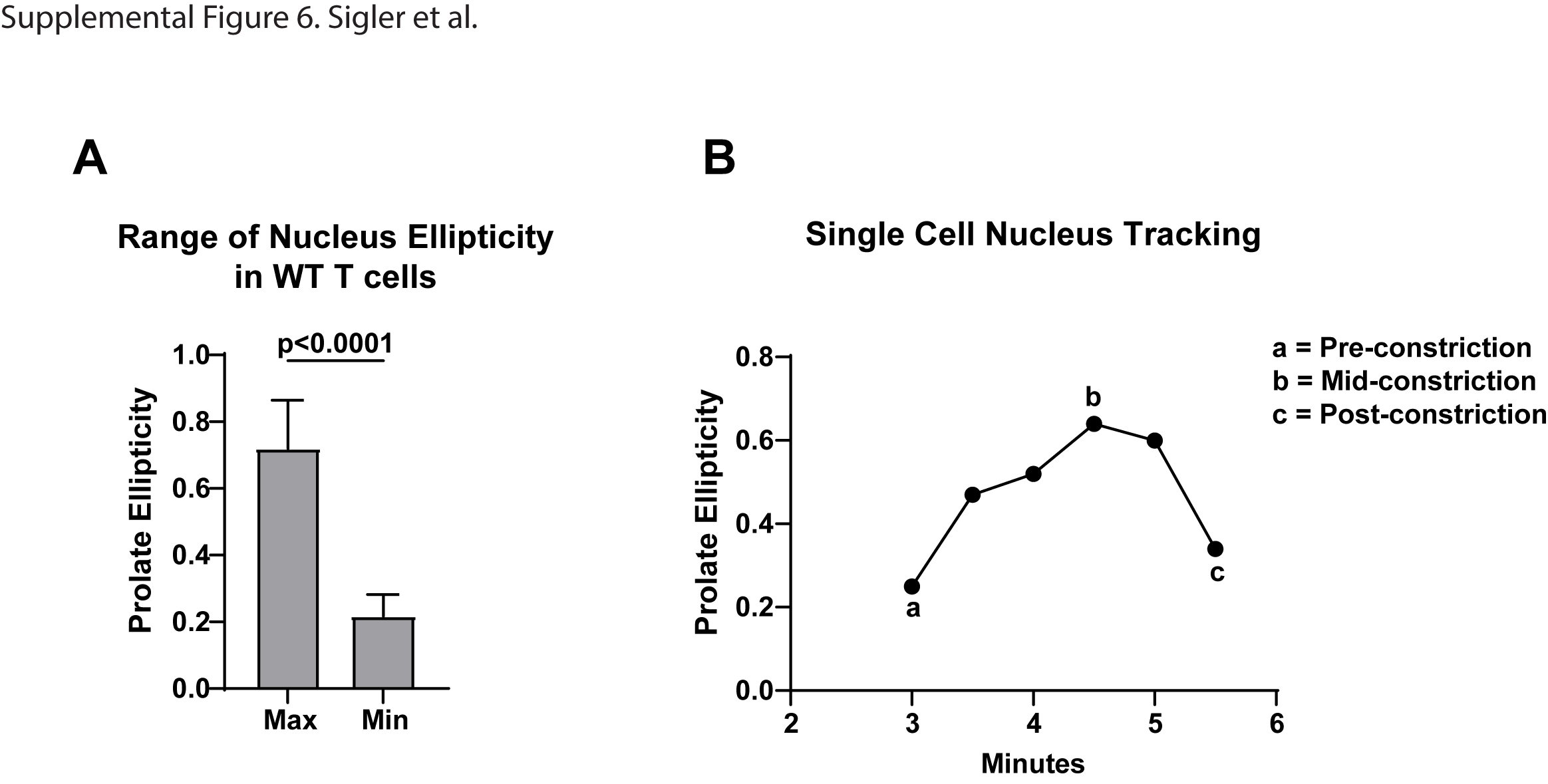

Supplement: Supplementary Figure 6 — Control T cells have a high dynamic range of nuclear ellipticity. Control T cells were collected from donor WT or LifeAct-GFP expressing donor mice and ex vivo activated. The cells were stained with Hoechst nuclear dye and embedded in 2.0 mg/mL collagen matrices seeded with fluorescent microspheres. T cell cortex and cytoplasm was visualized using either LifeAct-GFP or an additional CFSE dye labeling step. These data represent a subset of control cells from Figure 7 . (A) Maximum and minimum prolate ellipticity values of control cells migrating in 2.0 mg/mL collagen matrices. (B) Prolate ellipticity values over time for the control cell represented in Figure 7A . Pre-, Mid-, and Post-constriction points were determined by quantitative and qualitative features. Pre- and post-constriction points were determined by identifying local minima values of prolate ellipticity. The mid-constriction point was determined by the local maximum value of prolate ellipticity in combination with the image frame where the cell is maximally engaged with the constriction visualized by the fluorescent microspheres. Data in (A) represent the mean +/- SEM of 37 cells pooled from three independent experiments, including a subset of control cells used in Figure 7 . Significance was determined by unpaired t-test. [file Image6.tif]

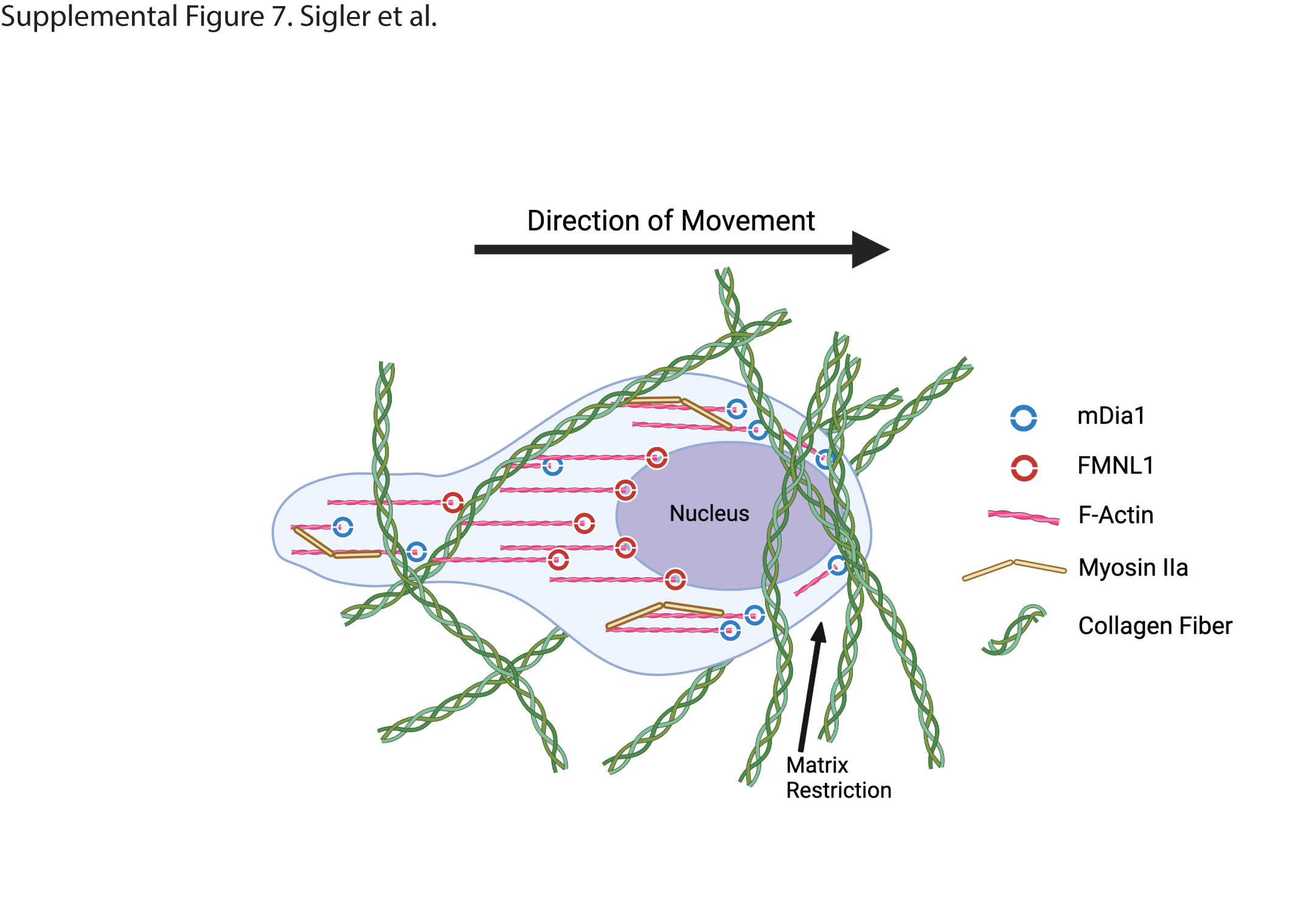

Supplement: Supplementary Figure 7 — Graphical model of formin-mediated T cell migration in 3D environments Illustration of a T cell encountering a restriction point in a 3D collagen environment. We propose a model of formin-mediated T cell migration in which FMNL1 and mDia1 have distinct roles. Our data suggests that FMNL1 is localized to the rear of the T cell and promotes deformation of the rigid nucleus independently of Myosin II via physical association with the nuclear compartment. Conversely, mDia1 is distributed more evenly throughout the cell and is almost entirely cytoplasmic, promoting general T cell motility in conjunction with Myosin II activity. Created with BioRender.com [file Image7.tif]
